# Higher general intelligence is associated with stable, efficient, and typical dynamic functional brain connectivity patterns

**DOI:** 10.1162/imag_a_00234

**Published:** 2024-07-17

**Authors:** Justin Ng, Ju-Chi Yu, Jamie D. Feusner, Colin Hawco

**Affiliations:** Campbell Family Mental Health Research Institute, Centre for Addiction and Mental Health, Toronto, ON, Canada; Institute of Medical Science, University of Toronto, Toronto, ON, Canada; Department of Psychiatry, University of Toronto, Toronto, ON, Canada; Department of Women’s and Children’s Health, Karolinska Institutet, Stockholm, Sweden

**Keywords:** g-factor, processing speed, resting-state fMRI, Human Connectome Project, dynamic functional connectivity, brain states

## Abstract

General intelligence, referred to as g, is hypothesized to emerge from the capacity to dynamically and adaptively reorganize macroscale brain connectivity. Temporal reconfiguration can be assessed using dynamic functional connectivity (dFC), which captures the propensity of brain connectivity to transition between a recurring repertoire of distinct states. Conventional dFC metrics commonly focus on categorical state switching frequencies which do not fully assess individual variation in continuous connectivity reconfiguration. Here, we supplement frequency measures by quantifying within-state connectivity consistency, dissimilarity between connectivity across states, and conformity of individual connectivity to group-average state connectivity. We utilized resting-state functional magnetic resonance imaging (fMRI) data from the large-scale Human Connectome Project and applied data-driven multivariate Partial Least Squares Correlation to explore emergent associations between dynamic network properties and cognitive ability. Our findings reveal a positive association between g and the stable maintenance of states characterized by distinct connectivity between higher-order networks, efficient reconfiguration (i.e., minimal connectivity changes during transitions between similar states, large connectivity changes between dissimilar states), and ability to sustain connectivity close to group-average state connectivity. This hints at fundamental properties of brain–behavior organization, suggesting that general cognitive processing capacity may be supported by the ability to efficiently reconfigure between stable and population-typical connectivity patterns.

## 1 Introduction

General intelligence measures the ability to excel across diverse situations, a capacity increasingly crucial for success in the modern world. It is quantifiable—positive correlations exist across cognitive test scores, indicating that proficiency in one area predicts competence in others (Spearman, 1961). The g-factor (g) measures this capacity and can be estimated by considering the underlying relationships across cognitive tests (Gignac & Bates, 2017). Lower levels link to adverse outcomes such as incarceration and poverty (Gottfredson, 2002,^2003^), whereas higher levels are associated with physical and mental well-being, academic and professional achievement, and even lifespan (Terman, 1954;Whalley & Deary, 2001). Furthermore, genetic studies suggest that effects span generations (Panizzon et al., 2014).

Given that g assesses shared performance across cognitive domains, it is hypothesized to be driven by shared neurobiological mechanisms (Barbey, 2018). Several theories emphasize brain networks. Parieto-Frontal Integration Theory (PFIT) underscores a network of frontal and parietal regions responsible for integrating and evaluating information (Jung & Haier, 2007). Similarly, Multiple Demand Theory (MD) highlights frontoparietal (FPN) and cingulo-opercular (CON) intrinsic connectivity networks (ICNs) which are commonly recruited to support diverse task demands through cognitive control (Crittenden et al., 2016;Duncan, 2010). Empirical studies support these ideas (Hilger et al., 2022), further implicating the dorsal attention (DAN) and default mode networks (DMN). Alternative theories emphasize whole-brain function. For instance, the neural efficiency hypothesis suggests that brain-wide efficiency underlies g (Neubauer & Fink, 2009). Efficiency can manifest in various forms, such as reduced energy expenditure or better communication between any two regions.

Another theory, Network Neuroscience Theory of Human Intelligence (Barbey, 2018), posits that g arises from the ability to flexibly reconfigure the whole-brain network in response to changing cognitive demands. Brain networks can be examined using functional connectivity (FC), defined as statistical associations between blood-oxygen-level-dependent (BOLD) signals detected by functional magnetic resonance imaging (fMRI) between brain regions. Traditionally, static FC (sFC) is computed using correlations over a scanning session. One avenue to explore this theory has been to examine the consistency of sFC between resting state (during no task) and task states. This reveals the phenomenon of “update efficiency,” where g positively associates with less required connectivity reconfiguration, aligning with the neural efficiency hypothesis (Schultz & Cole, 2016;Thiele et al., 2022;Xiang et al., 2022).

Another approach is to investigate dynamic FC (dFC) (Hutchison et al., 2013), which characterizes the evolution of FC during*t*time segments, or dFC(*t*), as opposed to the entire session. While prior research has linked cognitive performance to flexibility assessed at the level of connections and regions (Bassett et al., 2011;Braun et al., 2015;Jia et al., 2014), whole-brain network reconfiguration can also be quantified by clustering dFC(*t*) into a repertoire of recurring FC patterns to define “states” characterized by a single fundamental FC pattern, and examining transition frequency between states (Cabral et al., 2017).

We examined “frequency” metrics based onGirn et al.’s (2019)proposal: Stable maintenance of states characterized by low FC strength and corresponding high FC variability across classified dFC(*t*) should be associated with higher g based on prior associations with executive function (Nomi et al., 2017). This approach is further justified by the association between higher general intelligence and stability of network community structure across dFC(*t*) (Hilger et al., 2020), as well as links between state frequency metrics and diverse cognitive metrics, including cognitive integrity (Cabral et al., 2017), verbal reasoning, and visuospatial ability (Xia et al., 2019).

While two dFC(*t*) states classified to the same state have the same fundamental FC pattern, differences in the exact patterns are a source of individual variability ignored by frequency metrics. Distance metrics can quantify differences between dFC(*t*), with shorter distances representing greater similarity. Supporting its relevance,Battaglia et al. (2020)revealed positive correlations between total average dFC(t) distance and visuomotor performance. The study also showed that the dFC(*t*) trajectory can be classified into short (state maintenance) and distant jumps (state change), but did not separately explore distances within these attributes. Recent studies examining individual variability also suggest that better cognitive performance is associated with having more typical brain characteristics (Corriveau et al., 2023;Gallucci et al., 2022;Hahamy et al., 2015;Hawco et al., 2020). A typical characteristic may be beneficial because evolution favors population prevalence of advantageous traits. Optimal traits can be characterized by minimizing idiosyncratic imperfections through group averaging, like how the average face is attractive. Alternatively, cognitive proficiency may arise from unique adaptations. Idiosyncrasy has been quantified through discriminability (i.e., fingerprinting), which assesses how well a measure identifies an individual from the group. sFC discriminability increases throughout development and is delayed in individuals with higher psychiatric burden (Kaufmann et al., 2017), sFC transformations which enhance discriminability improve behavioral prediction (Amico & Goñi, 2018;Elliott et al., 2019), and sFC connections contributing most to discrimination predict fluid intelligence (Finn et al., 2015). However, brain features driving discriminability may not necessarily predict behavior (Byrge & Kennedy, 2020;Finn & Rosenberg, 2021;Mantwill et al., 2022;Noble et al., 2017), suggesting that typicality may not predict cognition at all. To further characterize whole-brain network reconfiguration, we propose “transition distance” metrics indexing dFC(*t*) distances during state maintenance and changes, and “idiosyncrasy” metrics indexing divergence of dFC(*t*) from group-average state patterns.

This study explores the association between g and network reconfiguration in the Human Connectome Project (HCP) dataset using Partial Least Squares Correlation (PLSC) (Wold, 1982), a multivariate technique which identifies data-driven latent variable pairs maximally capturing covariance between two matrices—cognitive tests and network reconfiguration metrics (McIntosh & Lobaugh, 2004;McIntosh et al., 1996). By including all cognitive tests in the PLSC, we investigate whether g arises as a property of network reconfiguration (latent variables with high weights for most cognitive tests) or whether specific cognitive domains emerge (latent variables with high weights for a subset) (Agelink van Rentergem et al., 2020). We hypothesize that higher g is associated with frequency, transition distance, and idiosyncrasy metrics which, as suggested by previous studies, could reflect qualities implicated by theories of intelligence, higher state stability, and higher state typicality. This study represents one of the first attempts to directly assess the brain–behavior relationship proposed in the Network Neuroscience Theory of Human Intelligence using a large-scale dataset, employing both traditional and novel dFC metrics to characterize network reconfiguration.

## 2 Materials and Methods

### 2.1 Participants and data

Data for the current study were obtained from the HCP1200 release which was granted ethics approval by the Washington University Institutional Review Board (S. M. Smith et al., 2013). The sample consisted of 950 young adults (male = 448; age = 28.65 ± 3.70; race = 722 White, 126 Black, 60 Asian, 23 multiple, 17 unknown, and 2 American Indian) with all 4 ICA-FIX preprocessed 3T resting-state fMRI scans, all 10 selected cognitive test scores, and an average relative FD scanner motion <.2 mm for each scan to exclude scans with high noise.

### 2.2 Cognitive domain scores

We included 10 cognitive tests used in a factor analysis conducted byDubois et al. (2018)on HCP data in the PLSC. We give a brief description for what each test measures inTable 1. These tests originate from the NIH Toolbox (http://www.nihtoolbox.org) and the Penn Computerized Neurocognitive Battery (Gur et al., 2010). Notably, CardSort and Flanker scores are based on a combination of accuracy and reaction time, while other test scores are generally derived from accuracy. Further test descriptions are located inDubois et al. (2018)and the source documents for corresponding cognitive tests.

**Table 1.** Cognitive test descriptions.

Dimensional change card sort (CardSort)	Cognitive flexibility
Flanker Inhibitory Control and Attention (Flanker)	Cognitive Control
List Sorting Working Memory (ListSort)	Working Memory
Picture Sequence Memory (PicSeq)	Visual Episodic Memory
Picture Vocabulary (PicVoc)	Picture-Based Vocabulary
Pattern Comparison Processing Speed (ProcSpeed)	Simple Processing Speed
Oral Reading Recognition (ReadVoc)	Reading-Based Vocabulary
Penn Progressive Matrices (PMAT24)	Fluid Ability
Penn Word Memory (WordMem)	Verbal Episodic Memory
Penn Line Orientation (LineOrient)	Spatial Orientation

### 2.3 fMRI collection and preprocessing

3T resting-state BOLD fMRI acquisition and preprocessing details can be found in the original publications (Salimi-Khorshidi et al., 2014;S. M. Smith et al., 2013). In brief, four scans (REST1_LR, REST1_RL, REST2_LR, REST2_RL) were collected over two sessions (one per day) and two 15 minutes runs that differed in phase-encoding direction (left-right and right-left). Individuals were instructed to lie still and fixate on a central cross. A multiband slice acquisition sequence was done using 3T MRI Siemens “Skyra” scanner (TR = 720 ms, TE = 33 ms, flip angle = 52^o^, voxel size = 2 mm isotropic, 72 slices at multiband acceleration factor = 8, 104 x 90 matrix). Selected surface-based data had undergone HCP minimal preprocessing (including B0 distortion correction, coregistration to T1-weighted images, and normalization to the surface template), ICA-FIX, and motion confound regression. ICA-FIX removes noise components of the signal such as motion, and performs well relative to motion correction methods such as volume censoring with less data loss (Parkes et al., 2018). Motion confound regression was conducted with 24 motion parameters (3 translational and 3 rotational terms, and the corresponding 6 quadratic terms, 6 temporal derivatives, and 6 quadratic expansions of the 6 temporal derivatives) to further minimize motion artifacts. BOLD voxel timeseries were demeaned within each voxel, then averaged within 360 cortical regions according to the HCP-MMP1.0 atlas (Glasser et al., 2016).

### 2.4 Leading Eigenvector Dynamics Analysis

We used Leading Eigenvector Dynamic Analysis (LEiDA) (Cabral et al., 2017) to generate phase dFC states while minimizing computational costs and avoiding the challenges of the traditional “sliding window” approaches (Preti et al., 2017) (seeFig. 1for an analysis flowchart). As with other forms of FC, high values are thought to represent better communication between regions. Phase dFC specifically relates to the communication through coherence theory (Fries, 2015), suggesting that dFC coherence values calculated instantaneously characterize the communication between regions occurring through phase synchronization. Phase dFC also has a natural correspondence with sFC. For example, dFC matrices averaged across time strongly resemble sFC (Appendix A).

**Fig. 1.**
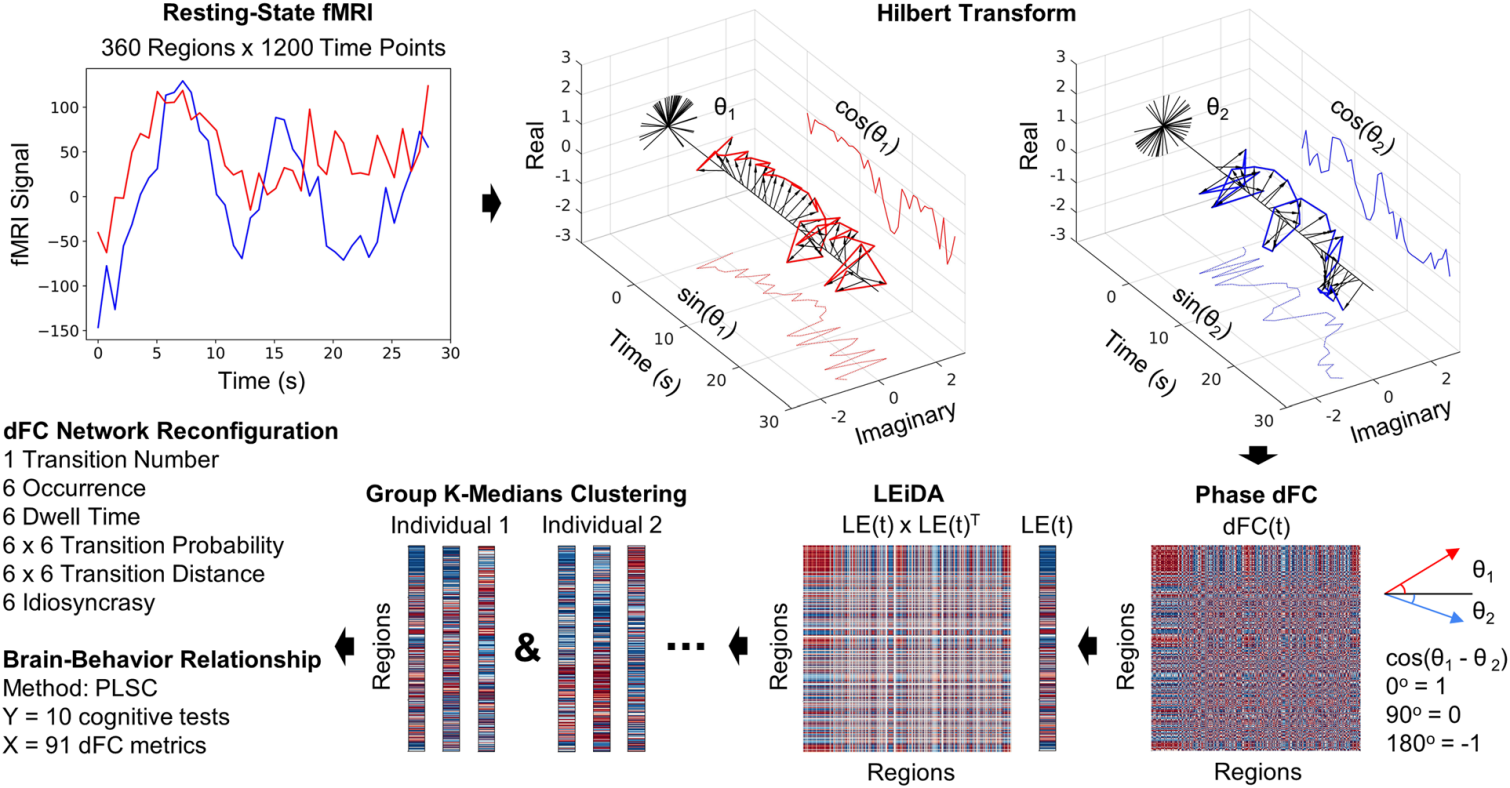
Analysis flowchart. Positive values are represented as red and negative values in blue within the matrices. We employed resting-state fMRI data. The Hilbert transform was utilized to convert the fMRI timeseries for each region into a revolution and retrieve phase angles at each*t*time point, enabling the calculation of phase differences between regions at each*t*time point between every region, resulting in dFC(*t*). We then extracted the leading eigenvector LE(*t*) to reduce dimensionality per LEiDA (Cabral et al., 2017). Subsequently, k-medians clustering with*k*= 6 was conducted on the concatenated LE(*t*) across individuals. Each individual’s clustering was employed to compute network reconfiguration metrics. To investigate the relationship between cognition and network reconfiguration, a multivariate PLSC analysis was conducted using 10 cognitive tests and all 91 dFC network reconfiguration metrics.

In LEiDA (Cabral et al., 2017), phase dFC analysis is conducted to calculate instantaneous dFC(*t*) at every time point. The phase*θ*(*n*,*t*) at each time point*t*in the*n*th region was derived from the region’s fMRI timeseries by the Hilbert transform, which expresses the regional signal*x*(*n*,*t*) as the product of the amplitude*A*and the phase*θ*, as*x*(*n, t*) =*A*(*n, t*)*θ(*n, t*). The phase*θ*adds a third “imaginary” axis (along with the real and time axes) that converts the rise and fall of fMRI signals into an angle (Cabral et al., 2017;Lord et al., 2019). The first and last time points were removed to exclude boundary artifacts induced by the Hilbert transform (Vohryzek et al., 2020). At each*t*, cosine of the phase difference between regions*n_1_*and*n_2_*quantifies the fMRI signal synchronization and thus FC, namely dFC(*n_1-2_*,*t*) = cos(*θ*(*n_1_*,*t*) -*θ*(*n_2_*,*t*)); positive values indicate coherence (1 = 0° difference), 0 value indicates orthogonality (0 = 90° difference), and negative values indicate anticoherence (-1 = 180° difference). Eigendecomposition of dFC(*t*) at each*t*was done to compress the 360 x 360 region phase connectivity matrix into its dominant pattern, represented by its first 360 region x 1 eigenvector (i.e., the leading eigenvector which captures the most original variance, with the largest eigenvalue), LE(*t*), to improve clustering performance and reduce computational cost. The sign of LE(*t*) loadings classifies regions into two communities—the coherent majority, and the minority of regions anticoherent with this majority. Given that signs are arbitrary after eigendecomposition, the sign of the coherent majority was set to negative, while the sign of the anticoherent minority was set to positive, to maintain consistency. LE(*t*) x LE(*t*)^T^visualizes the dominant 360 x 360 pattern at each*t*, which can be compared with the raw 360 x 360 dFC(*t*) pattern.

### 2.5 State identification

Clustering analysis was applied to LE(*t*) to classify*t*time points into discrete clusters referred to as states, where corresponding*t*has similar recurring fundamental connectivity patterns (Cabral et al., 2017).*k*-medians clustering with Manhattan distance (rather than Euclidean distance which has worse performance for high-dimensional data) was conducted on concatenated time points from all runs and individuals with 500 repetitions to escape local minima (Aggarwal et al., 2001;Allen et al., 2014). The median of the LE(*t*) in each state represents the fundamental dominant connectivity pattern across all*t*within the state, referred to as the LE state. The average of all dFC(*t*) in each state according to the LE(*t*) clustering represents the fundamental raw connectivity pattern, referred to as the dFC state.

**Fig. B.1.**
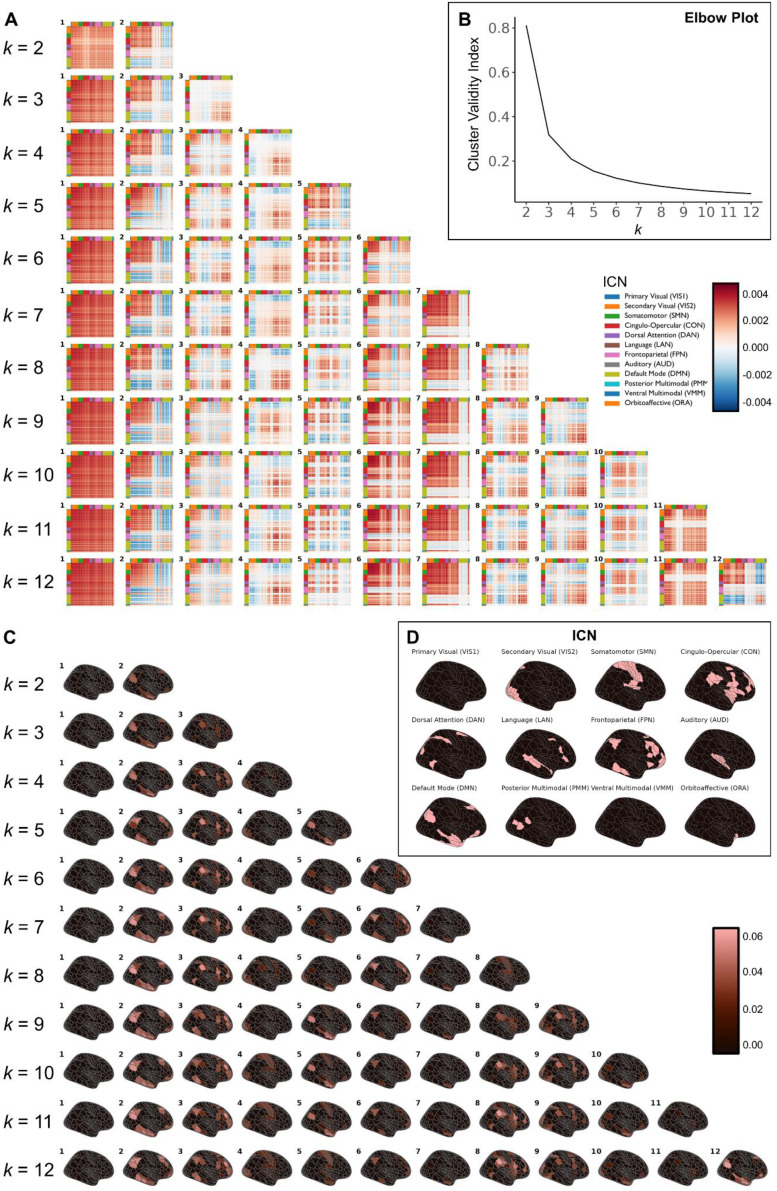
LE matrices and brain plots for*k*= 2-12. To choose*k*for the main analysis, we conducted*k*-medians clustering across*k*= 2-12. The median LE x LE^T^patterns are displayed, where number labels correspond to similar states across*k*(A). Red refers to positive coherence, white refers to zero coherence, and blue refers to negative coherence. In line with prior studies (Nomi et al., 2017), we investigated the elbow plot with the cluster validity index (ratio comparing within- and between-cluster distances) (B). The elbow value seemed to range from*k*= 4-5, which matches*k*commonly used in similar analyses (Allen et al., 2014;Cabral et al., 2017;Damaraju et al., 2014;Nomi et al., 2017). We also plotted the positive values of the median LE in each region to show regions with opposite phases from the rest (Vohryzek et al., 2020), with numbers labeling visually similar states across*k*(C). As expected, the resting-state sFC-derived ICNs were delineated. We also plotted the Cole-Anticevic ICN parcellations (Ji et al., 2019) to give context (D).

The choice of*k*determines the number of states. We considered*k*= 2-12 (Appendix B). Similar analyses choose*k*= 4-5 based on the elbow method, a heuristic to choose a*k*that matches the number of true clusters without cluster subdivision (Allen et al., 2014;Cabral et al., 2017;Damaraju et al., 2014;Nomi et al., 2017). In line with this, we depict similar results for the elbow method (Fig. B.1B). However, the brain does not necessarily enter connectivity patterns segregated between a small number of discrete clusters as assumed by the elbow method. Instead, different lower-order representations associated with different*k*can be used to better understand brain function (Figueroa et al., 2019;Lord et al., 2019). Each state commonly displays at least one ICN anticoherent with the rest of the brain (Vohryzek et al., 2020), delineating it, represented by positive loadings for corresponding ICN regions in the median LE state (Fig. B.1C). This was viewed with the median LE x LE^T^state and the plot of the positive loadings on the brain (Fig. B.1A). We chose*k*= 6 for the main analysis, as it is close to the commonly employed*k*= 4-5, but includes a state delineating the FPN (i.e., anticoherent) which may be particularly important for g (Duncan, 2010;Jung & Haier, 2007). We also repeated the main analysis for*k*= 2-12 to examine whether the main findings replicate across*k*post hoc.

### 2.6 State frequency

To assess state frequency metrics, we used state clustering within runs to calculate 6 stateoccurrences—proportion of*t*classified to each state (*how often the state occurs*); 6dwell times—average number of*t*classified to a given state of the six before switching (*how long the state is maintained*); 1transition number—total number of state switches (*how often states switch*); and 6 x 6transition probabilities—proportion of each state switch (e.g., States 1 to 2), including switching to itself (i.e., staying in the state), to all state switches starting from the same state (e.g., all state switches from State 1) (*how often this state switch occurs*). Each metric was averaged across runs.

### 2.7 State transition distance

We calculated 6 x 6 statetransition distanceswhich measure the average distance between LE(*t*) during a given state switch (*how much connectivity changes during this state switch*). Formally, the Manhattan distance between all sequential pairs of LE(*t*) in each run was calculated. Each LE(*t*) pair was categorized as a specific state switch. The mean distance was computed across all instances of a state switch and averaged across runs.

### 2.8 State idiosyncrasy

We calculated 6 stateidiosyncrasymetrics which measure the average distance between LE(*t*) and the group LE median of its state (*how different are connectivity patterns from their state’s group average*). Formally, the Manhattan distance between each individual LE(*t*) and the group median of the corresponding LE state was calculated and averaged across runs for each state.

### 2.9 Statistical analysis

#### 2.9.1 Multivariate PLSC

We explored the multivariate association between 91 network reconfiguration characteristics (49 frequency, 36 transition distance, and 6 idiosyncrasy metrics) and 10 cognitive variables (10 cognitive tests) using PLSC (Krishnan et al., 2011). Age and gender were regressed from both sets of variables prior to statistical analysis to control for their effects without compromising sample size and generalizability (Agelink van Rentergem et al., 2020). Sex was not used because HCP only provides self-reported gender. To allow comparison across measures, variables were also standardized by computing their*Z*-scores. As the proportion of related participants in the Human Connectome Project is high (Van Essen et al., 2013), we also accounted for family structure in statistical analyses.

PLSC extracts pairs of new variables, called latent variables, constructed by linear combination of original variables within two matrices (i.e., cognitive tests and network reconfiguration metrics). Each pair of latent variables (i.e., cognition and network reconfiguration) comprises a dimension which maximally captures the overall covariance between the matrices, with the constraint that subsequent dimensions are orthogonal (i.e., a latent variable from one matrix is uncorrelated with the latent variable from the other matrix in other dimensions) from previously constructed dimensions. This is accomplished via singular value decomposition, which generates singular values (SVs) representing covariance for each dimension. The Spearman’s correlation between cognition and network reconfiguration latent variables can also be used to provide a standardized assessment of association strength (Ziegler et al., 2013), complementing the relative association strength represented by SV. Dimensions are organized according to the proportion of covariance between the two input matrices explained. Importantly, because dimensions are identified via covariance across both matrices, dimensions are driven by the relationships across these matrices—that is, each dimension represents a data-driven reflection of a brain–behavior relationship. In our case, PLSC could identify relationships reflective of generalized cognition and/or of specific cognitive domains that share common network characteristics.

Each cognition and network reconfiguration latent variable consists of scores for individuals, indicating their position along the dimension. Different weights are applied in a linear combination of the original variables to construct each latent variable. Loadings can be derived from the covariance between each original variable and the latent variable which facilitate interpretation by quantifying the relative contributions of cognition and network reconfiguration characteristics driving the dimension. For more details, seeKrishnan et al. (2011).

##### 2.9.1.1 Reliability of PLSC dimensions

Nonparametric permutation tests on SVs were used to examine whether dimensions are significant (McIntosh & Lobaugh, 2004), indicating that they are reliable. Specifically, after confound regression and standardization, we permuted every variable in the cognitive and network reconfiguration matrices and computed the null distribution of SVs for every dimension. We accounted for family structure by generating blocks of unrelated individuals and solely permuting within those blocks alone (Ooi et al., 2022). This procedure was iterated 10,000 times. If*p*< .05, we considered the SV, thus the dimension, significant (*α*= .05), and reliable.

##### 2.9.1.2 Stability of PLSC loadings

To identify variables that contribute stably, we also used nonparametric bootstrapping to estimate the stability of the cognition and network reconfiguration PLSC loadings for each dimension (McIntosh & Lobaugh, 2004). Specifically, after confound regression and standardization of variables, we randomly sampled individuals with replacement and repeated PLSC to calculate bootstrapped loadings. We accounted for family structure by sampling entire blocks of related individuals with replacement (Field & Welsh, 2007;Ren et al., 2010). This procedure was iterated 10,000 times to generate bootstrap ratios (BRs) for each loading. We used a critical value of 2.5 (equals, approximately, the critical value at*α*= .01 for a two-tail*Z*-test) to determine whether the loading is stable. A critical value of 2 equals approximately the critical value at the frequently used*α*= .05 for a two-tail*Z*-test, but studies commonly use more stringent thresholds such as 2.5 (Mareckova et al., 2020;McCormick & Maguire, 2021;Zimmermann et al., 2018) or 3 (equals, approximately, the critical value at*α*= .001 for a two-tail*Z*-test) (Kardan et al., 2023;Lee et al., 2020;Ziegler et al., 2013) to determine stability. We chose to examine 2.5 to preserve more statistically stable loadings for interpretation of the PLSC latent variables, but we also used 3 as the critical value post hoc.

##### 2.9.1.3 Reproducibility of PLSC dimensions and latent variables

To assess the reproducibility of SVs, we performed split-half PLSC (Churchill et al., 2013,^2016^;McIntosh, 2021). In each iteration, we conducted random split-half sampling of individuals, set one half as a train set and one half as a test set in a train-test approach, and conducted singular value decomposition on both halves. We accounted for family structure by making sure that related individuals were not separated between splits (Ooi et al., 2022). We also accounted for data leakage by conducting confound regression and standardization on the test set using coefficients, mean, and standard deviation (*SD*) derived from the train set. We evaluated the reproducibility of dimensions across halves by deriving test SVs from using the latent variable pairs of the first train half and the correlation matrix of the second test half. Higher SVs mean higher reproducibility of the dimension. We evaluated the reproducibility of each set of latent variables (i.e., network reconfiguration or cognition) across halves using the similarity between the latent variables of the first and second halves as quantified by the dot product. Higher dot products indicate greater reproducibility of the latent variables. We repeated split-half resampling 10,000 times. For each dimension, the reproducibility score was calculated by approximating a*Z*-score—the mean of “test” SVs divided by the*SD*. For each latent variable set (i.e., network reconfiguration or cognition), the reproducibility score was also calculated by approximating a*Z*-score—the mean of the dot product between the latent variables across the two halves divided by the*SD*. Values greater than 1.95 were used to determine reproducibility (approximately 95% confidence interval).

##### 2.9.1.4 Cross-validation for PLSC

The reproducibility analysis compares PLSC outputs calculated from train and test set halves to quantify out-of-sample performance. Another way to quantify out-of-sample performance is to compare the original in-sample PLSC and a cross-validated out-of-sample PLSC summarizing multiple test sets. The in-sample PLSC tends to overestimate the true association strength and out-of-sample PLSC tends to underestimate the true association strength (Helmer et al., 2024). As we calculated the in-sample association strength, we bound our estimate by also calculating the out-of-sample PLSC association strength from cross-validation using Spearman’s correlation. The stability of PLSC latent variables across samples can also be assessed through a different approach in this framework. While we estimated the reproducibility of PLSC latent variables across samples, we additionally estimated the consistency of PLSC latent variables estimated from cross-validation and the original PLSC latent variables for comparison via Spearman’s correlation. More details can be found inAppendix C.

##### 2.9.1.5 Variance explained for PLSC

To contextualize how well our model explains the original data, we also quantified the variance of the original variables explained by PLSC latent variables. We additionally quantified out-of-sample variance explained using the test sets from the reproducibility and cross-validation analyses. More details can be found inAppendix C.

#### 2.9.2 Normality

Normality was checked for bivariate analyses using bivariate quantile-quantile plots and bivariate normality tests from the MVN package (Korkmaz et al., 2014). There was evidence against normality in many cases, justifying our choice of Spearman’s correlation over Pearson’s correlation across applicable analyses.

#### 2.9.3 Outliers

Outliers were not stringently checked for and excluded given that outliers may be considered true observations (i.e., under the influence of the same brain processes). Scatterplots for each bivariate analysis gave no visual indication that outliers were driving associations.

#### 2.9.4 Multiple comparisons

We corrected for multiple comparisons using Benjamini–Hochberg FDR-correction (Benjamini & Hochberg, 1995) where applicable for mass-univariate analyses. No correction was performed in relation to multivariate PLSC, consistent with prior studies (Kardan et al., 2023;Lee et al., 2020;Mareckova et al., 2020;McCormick & Maguire, 2021;Ziegler et al., 2013;Zimmermann et al., 2018). As the permutation test is done on the full PLSC model, multiple comparisons correction across dimensions is not necessary (Mareckova et al., 2020). No multiple comparisons correction is necessary for the bootstrapping analysis because no statistical test is performed (McIntosh & Lobaugh, 2004).

## 3 Results

### 3.1 dFC states characterize ICNs

**Fig. 2.**
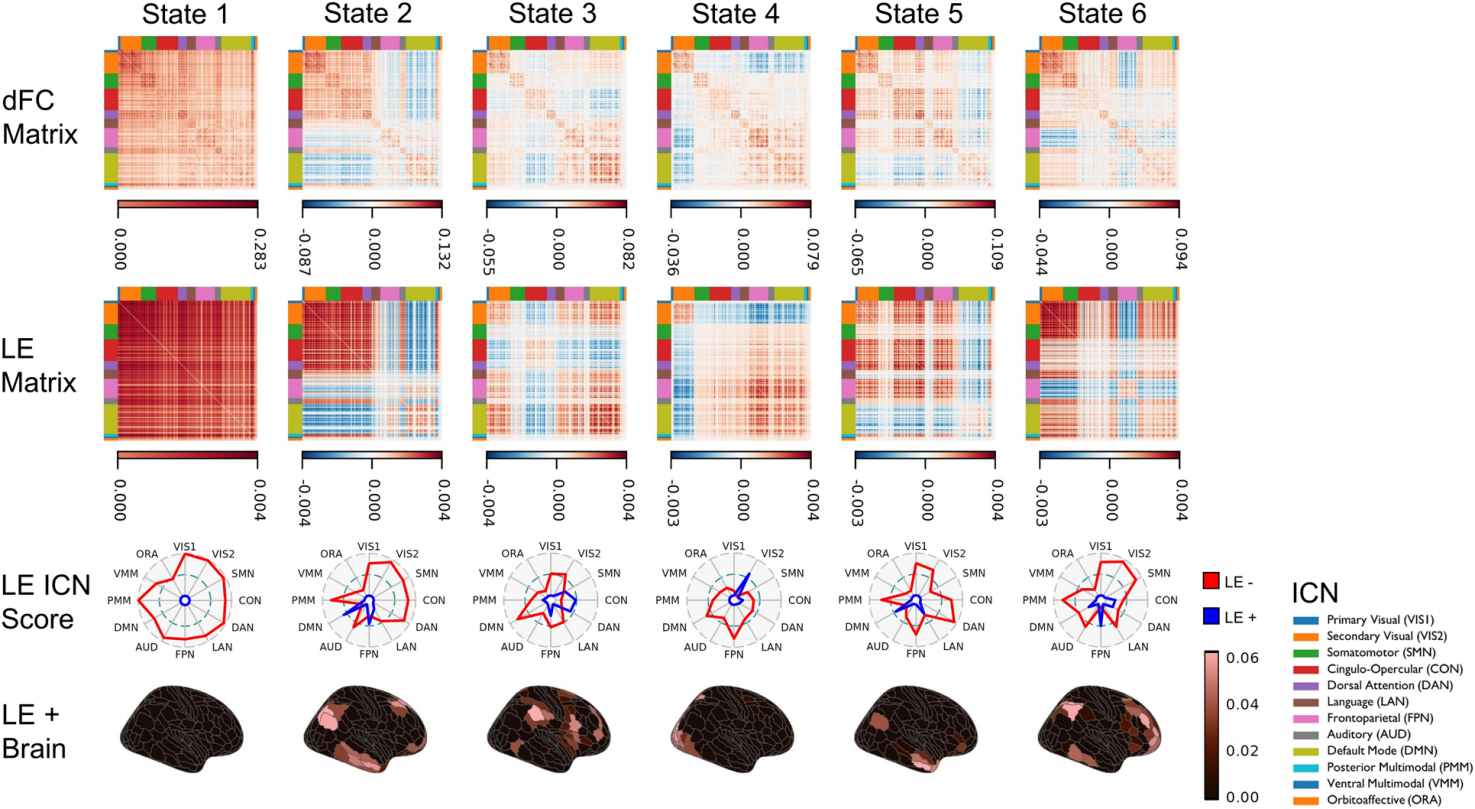
dFC states. Each matrix in the first and second rows is represented by separate value ranges to aid visual interpretation. Coherence is denoted in red, zero coherence in white, and anticoherence in blue. The first row shows group dFC states, visualized by averaging dFC(*t*) for*t*time points categorized into respective states through LE(*t*) clustering. The second row displays median LE states, the dominant patterns of corresponding dFC states, visualized by multiplying state medians of LE(*t*) by their transpose (LE x LE^T^). The third row exhibits radar plots which characterize the overall phase coherence of ICNs using region values in the median LE states. LE(*t*) divides regions into two communities based on phase coherence (i.e., positive and negative sign), where the larger community is designated the main coherence orientation (negative) and the smaller community is labeled as anticoherent to the main orientation (positive). ICN coherence scores are determined by averaging negative region values for each ICN (LE-, red), while ICN anticoherence scores are calculated by averaging positive region values (LE+, blue). In the fourth row, values on the brain equal to or below 0 are represented in black, while positive values are shown in red. This row depicts positive region values of the median LE states, delineating ICNs demonstrating anticoherence. Notably, State 1 lacks ICNs exhibiting anticoherence, distinguishing it from other states. This is one of several reasons why State 1 has been interpreted as a “meta-stable” state returned to after entering other states (Cabral et al., 2017;Vohryzek et al., 2020).

In the main analysis with*k*= 6 (Fig. 2), State 1 exhibited uniform phase direction, while other states displayed anticoherence. State 2 was characterized by CON-DAN coherence, DMN-FPN coherence, and anticoherence of DMN and FPN with other ICNs. State 3 was characterized by FPN-DMN coherence, CON-DAN coherence, and anticoherence of CON and DAN with other ICNs. State 4 was characterized by FPN-DMN coherence and anticoherence of the secondary visual network (VIS2) with other ICNs. State 5 was characterized by coherence of CON and DAN with FPN, and anticoherence of DMN with other ICNs. State 6 was characterized by anticoherence of FPN with other ICNs.

### 3.2 dFC states exhibit different FC strength and FC variability

We examined the hypothesis that maintaining states with low FC strength and corresponding high FC variability is associated with higher g (Girn et al., 2019;Nomi et al., 2017) (Appendix D). First, we verified the FC strength and FC variability correspondence by calculating FC strength and FC variability across time after concatenating all dFC(*t*). The Spearman’s correlation was -.96, confirming that connections with high strength have low variability, and vice versa. Next, we identified candidate states by calculating FC strength and FC variability across time after concatenating dFC(*t*) within each state. States 3 and 4 displayed connections with the lowest strength and highest variability, suggesting that maintaining these states could be associated with higher g.

### 3.3 Network reconfiguration metrics exhibit distinct variability

Salient trends emerge in metric distributions across individuals (Appendix E; alsoFig. 4). Within-state transition probability (probability of remaining in the state) was highest and within-state transition distance (transition distance to the same state) was lowest compared with other transitions. This is expected because connectivity patterns were classified to the same state based on FC similarity. Transition distance distributions were comparable for reciprocal transitions (e.g., 1-2 and 2-1). Similarity is expected due to theoretical equivalence of distances between the fundamental connectivity patterns. Notably, State 1 stands out with the highest occurrence, dwell time, within-state transition probability, and target transition probability (transition probability toward that state) compared with other states. This observation and its uniform phase direction lend to why State 1 has been interpreted as a meta-stable state returned to after entering other states (Cabral et al., 2017;Vohryzek et al., 2020).

To further characterize the metrics, we also assessed the test–retest reliability (i.e., stability of the metric across repeated tests) across two different days using the Intraclass Correlation Coefficient (ICC) to characterize how well each metric represents a stable “trait” in an individual (Appendix F). Frequency metrics exhibited similar ICC to prior work (Choe et al., 2017;Li et al., 2020;D. M. Smith et al., 2018;Vohryzek et al., 2020;Zhao et al., 2019). All reconfiguration metrics displayed positive ICC, indicating that they can distinguish individuals from a group (Vohryzek et al., 2020). Notably, frequency metrics involving State 1, within-state transition distance metrics, and idiosyncrasy metrics all exhibited particularly high ICC.

Given that both traditional frequency-based and novel distance-based metrics such as transition distance/idiosyncrasy are calculated from dFC, we also assessed whether novel distance-based metrics capture the same variance as traditional frequency metrics based on correlations (Appendix G). We find that individual variability characterized by distance-based metrics was not fully identical to frequency-based metrics.

### 3.4 PLSC describes two significant and reproducible latent dimensions

**Fig. H.1.**
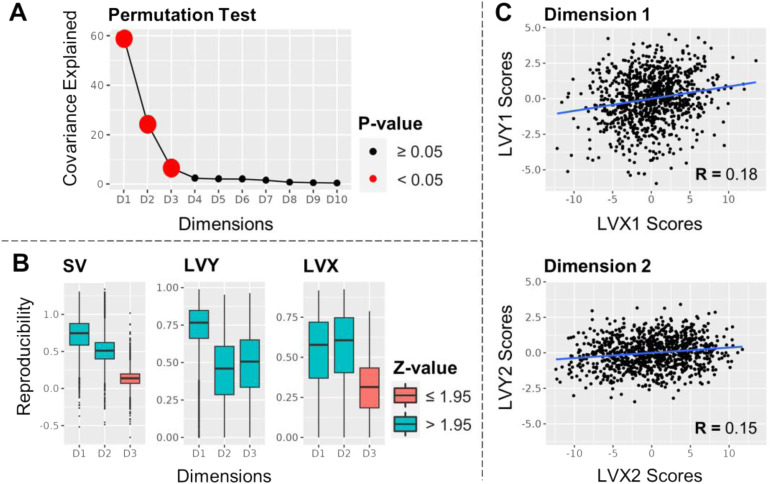
PLSC Diagnostics. Results for the permutation test with permuted blocks respecting family structure are plotted in the first row on the left (A). The y**-**axis describes the total covariance explained by the SV. D refers to the dimensions. Results for the reproducibility analysis with splits respecting family structure are plotted in the second row on the left (B). The reproducibility scores for singular values, latent variables of**Y**(LVY), and latent variables of**X**(LVX) are plotted from left to right. The Spearman’s correlation and scatter plots of scores for the latent variables of**Y**(LVY) and latent variables of**X**(LVX) of the first and second dimensions are plotted on the right (C). Covariance explained, SV values,*p*-values for the permutation test, SV reproducibility*Z*values, LVY reproducibility*Z*values, LVX reproducibility*Z*values, and Spearman’s correlations for the latent variable scores are located inSupplemental Table 2.

Permutation tests on SVs were utilized to assess the significance (*p*< .05) of the covariance in each dimension (McIntosh & Lobaugh, 2004). Dimensions 1 through 3 demonstrated significant proportions of covariance (Fig. H.1AinAppendix H). Specifically, Dimension 1 explained 58.83% (SV = 1187,*p*< .0001) of total covariance between network reconfiguration and cognition variables. Dimensions 2 and 3 accounted for 24.24% (SV = 762,*p*< .0001) and 6.57% (SV = 397,*p*< .0001), respectively. A split-half procedure was employed to assess reproducibility (*Z*> 1.95) of the SV and latent variables in each dimension (Churchill et al., 2013,^2016^;McIntosh, 2021). This analysis indicated that Dimensions 1 (SV*Z*= 3.22, network reconfiguration*Z*= 2.35, cognition*Z*= 4.40) and 2 (SV*Z*= 2.69, network reconfiguration*Z*= 2.50, cognition*Z*= 2.08) were reproducible, while Dimension 3 was not (SV*Z*= 1.16, network reconfiguration*Z*= 1.91, cognition*Z*= 2.25) (Fig. H.1B). Consequently, our analysis focuses on Dimensions 1 and 2.

Complementary to the reproducibility analysis for the latent variables, we assessed the consistency between the original latent variables and the estimated latent variables derived from a 10-fold cross-validation. These latent variables are highly correlated with Spearman’s correlations of .98 for the network reconfiguration latent variables and .99 for the cognition latent variables for Dimension 1, and .99 for the network reconfiguration latent variables and .96 for the cognition latent variables for Dimension 2, confirming the consistency across resampling.

In comparison with SVs, correlations between latent variables provide a standardized assessment of association strength. The Spearman’s correlation was .18 for Dimension 1 and .15 for Dimension 2 (Fig. H.1C). However, in-sample association strength tends to overestimate the true association strength (Helmer et al., 2024), so we also calculated out-of-sample association strength from cross-validation. The Spearman’s correlation was .12 for Dimension 1 and .13 for Dimension 2.

We also evaluated how well latent variables from PLSC could explain the original variables. Original PLSC latent variables explained 8.79% of network reconfiguration variance and 11.3% of cognition variance, reproducibility test set latent variables explained 10.2% of network reconfiguration variance and 12.0% of cognition variance on average, and cross-validation test set latent variables explained 10.0% of network reconfiguration variance and 12.0% of cognition variance on average, suggesting a relatively low but consistent in-sample and out-of-sample variance explained.

#### 3.4.1 Dimension 1 reflects relationships between g and network reconfiguration

**Fig. 3.**
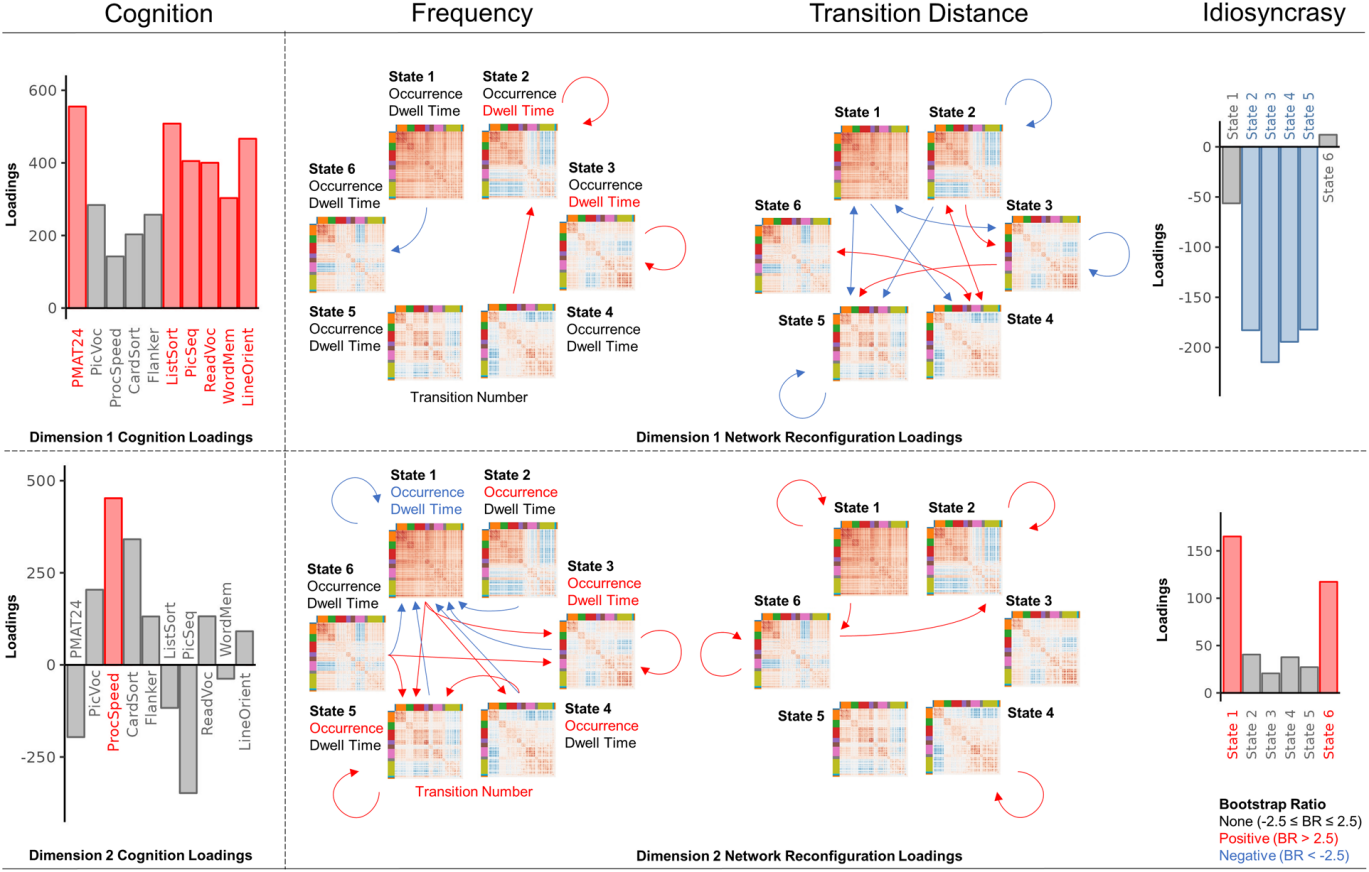
PLSC relationships between cognition and network reconfiguration metrics. We employed PLSC to assess the relationship between cognition and network reconfiguration. This involved creating pairs of latent variables representing cognition and network reconfiguration through a linear combination of 10 cognitive test variables and 91 network reconfiguration variables. The 10 cognitive tests included Dimensional Change Card Sort (CardSort), Flanker Inhibitory Control and Attention (Flanker), List Sorting Working Memory (ListSort), Picture Sequence Memory (PicSeq), Picture Vocabulary (PicVoc), Pattern Comparison Processing Speed (ProcSpeed), Oral Reading Recognition (ReadVoc), Penn Progressive Matrices (PMAT24), Penn Word Memory (WordMem), and Variable Short Penn Line Orientation (LineOrient). Using bootstrapping, we assessed the contributing loadings (i.e., weights) for original variables on latent variables. Stable positive bootstrap ratios (|BR| >2.5) are represented in red, while stable negative bootstrap ratios are in blue. The arrows refer to transition probabilities for frequency metrics and transition distances for transition distance, with circular arrows denoting within-state transitions or distances. Each dimension refers to a unique pair of cognition and network reconfiguration latent variables. For the Dimension 1 cognition loadings, the majority for positive and displayed stability, suggesting it represents g. In contrast, for Dimension 2 cognition loadings, only processing speed displayed stability. PLSC loading values and bootstrap ratios are located inSupplemental Table 3. A more stringent threshold with |BR| >3 was also conducted, with largely consistent results (Fig. H.2).

Bootstrapping on loadings was used to identify variables with stable (BR > 2.5) weighting (McIntosh & Lobaugh, 2004). In Dimension 1, all cognitive tests loaded in the same direction, characterizing a general measure of cognitive performance akin to g. Several cognitive tests exhibited stable loadings (PMAT24, ListSort, PicSeq, ReadVoc, WordMem, LineOrient; BR = 4.26, 3.90, 3.13, 3.20, 2.51, 3.53) but not all (Fig. 3). Notably, variables without stable loadings, with the exception of PicVoc, were tests accounting for reaction time.

##### 3.4.1.1 Frequency relates to g in Dimension 1

Dimension 1 exhibited stable positive loadings for dwell time and within-state transition probability of States 2 and 3 (dwell 2, dwell 3, 2-2, 3-3; BR = 2.86, 2.51, 3.08, 2.55) and target transition probability for State 2 (4-2; BR = 2.50) (Fig. 3). Additionally, Dimension 1 exhibited a stable negative loading for target transition probability for State 6 (1-6; BR = -3.20). In other words, primarily stability (frequent classification of subsequent LE(*t*) to the same state) in States 2 and 3, and lower frequency of State 6.

##### 3.4.1.2 Transition distance relates to g in Dimension 1

**Fig. 4.**
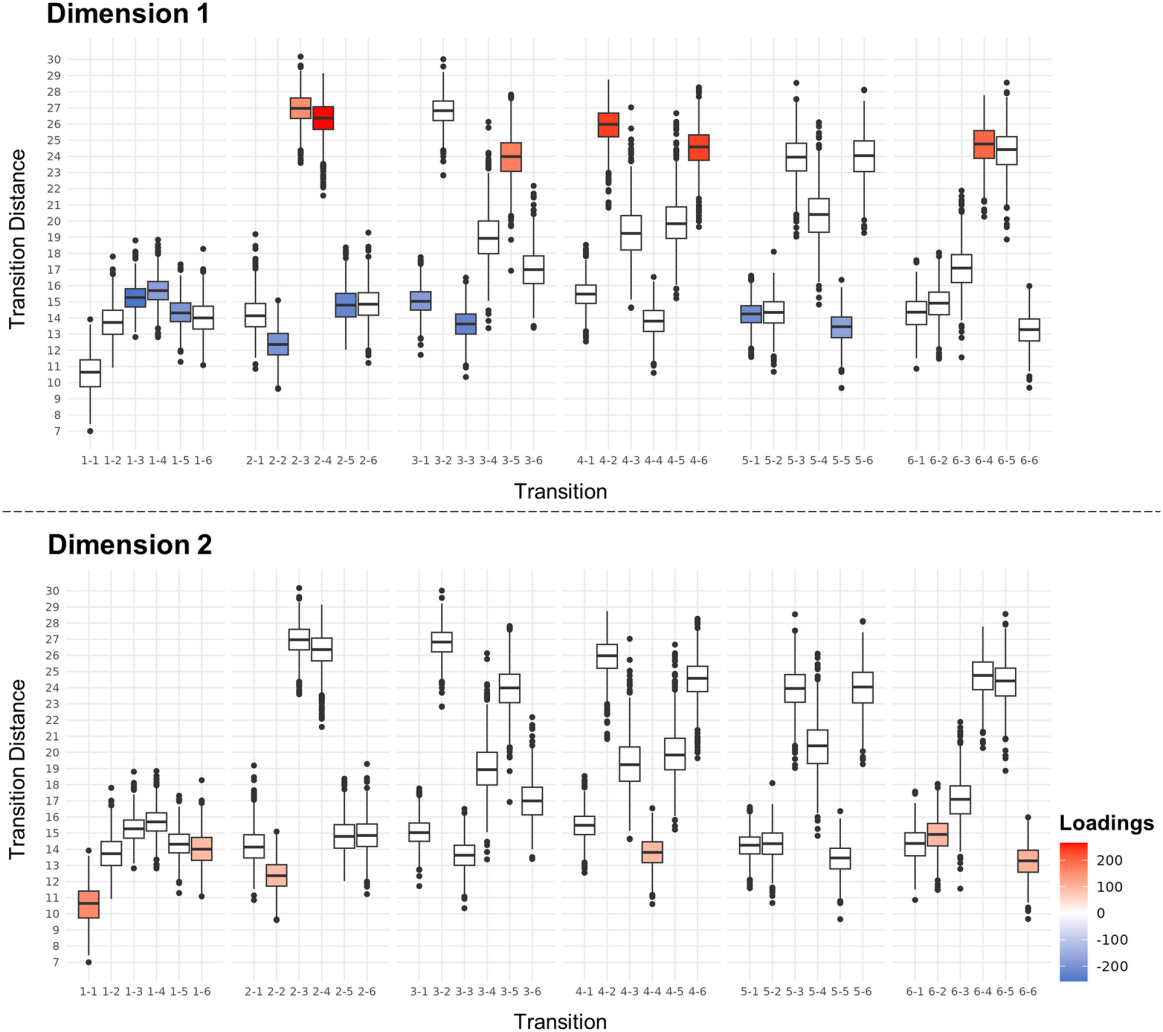
Divergence between distant and nearby transitions. The box plots represent transition distances within and between states across individuals. Stable (|BR| >2.5) positive bootstrap ratios are denoted in red, stable negative bootstrap ratios in blue, and unstable bootstrap ratios in white. The association between g and network reconfiguration was positive for transitions with higher transition distance values compared with other transitions (Dimension 1), and negative for transitions with lower transition distance values. Conversely, processing speed and network reconfiguration exhibited a positive relationship for transitions with lower transition distance values compared with other transitions (Dimension 2).

Stable (|BR| >2.5) relationships between transition distance and cognition were generally mirrored for reciprocal transitions (e.g., 1-2 vs. 2-1) (Fig. 3), suggesting similar importance for transition distances in both directions. Dimension 1 demonstrated stable positive loadings for transition distances between states (2-3, 2-4, 4-2, 3-5, 4-6, 6-4; BR = 2.50, 4.46, 3.92, 3.07, 4.09, 3.60) which have high transition distances across individuals compared with other transitions (Fig. 4), indicating dissimilar states. Conversely, Dimension 1 exhibited stable negative loadings for transition distances within state in States 2, 3, and 5 (2-2, 3-3, 5-5; BR = -3.21, -3.80, -2.81), between State 1 and others (1-3, 3-1, 1-4, 1-5, 5-1; BR = -4.43, -3.29, -3.32, -3.19, -3.51), and between States 2 and 5 (2-5; BR = -3.68) which have low transition distances (Fig. 4), indicating similar states. This implies efficiency, with larger transition distances for transitions between dissimilar states and smaller transition distances between similar states, including within-state transitions. The latter observation reinforces associations with stability (sequential LE(*t*) similarity).

##### 3.4.1.3 Idiosyncrasy relates to g in Dimension 1

Dimension 1 displayed stable (|BR| >2.5) negative idiosyncrasy loadings for States 2 through 5 (idiosyncrasy 2, 3, 4, 5; BR = -2.81, -3.59, -3.16, -3.05) (Fig. 3), implying typical connectivity. This also characterizes another form of stability (similarity of LE(*t*) to the state center) linked to g.

##### 3.4.1.4 Within-state stability, between-state reconfiguration efficiency, and state idiosyncrasy interrelate

Combining observations, we propose that higher g is associated with efficient reconfigurations between consistent connectivity patterns within states which are close to the state’s group average. This is contingent on characteristics coexisting, so we examined correlations between summary measures (details inAppendix I). Spearman’s correlations were significant (False Discovery Rate or FDR-corrected*p*< 1 × 10^-17^) and generally high (Table I.1), supporting that individuals with consistent connectivity patterns also tend to reconfigure efficiently and have typical patterns.

##### 3.4.1.5 Psychometric g reveals consistent results

**Fig. J.1.**
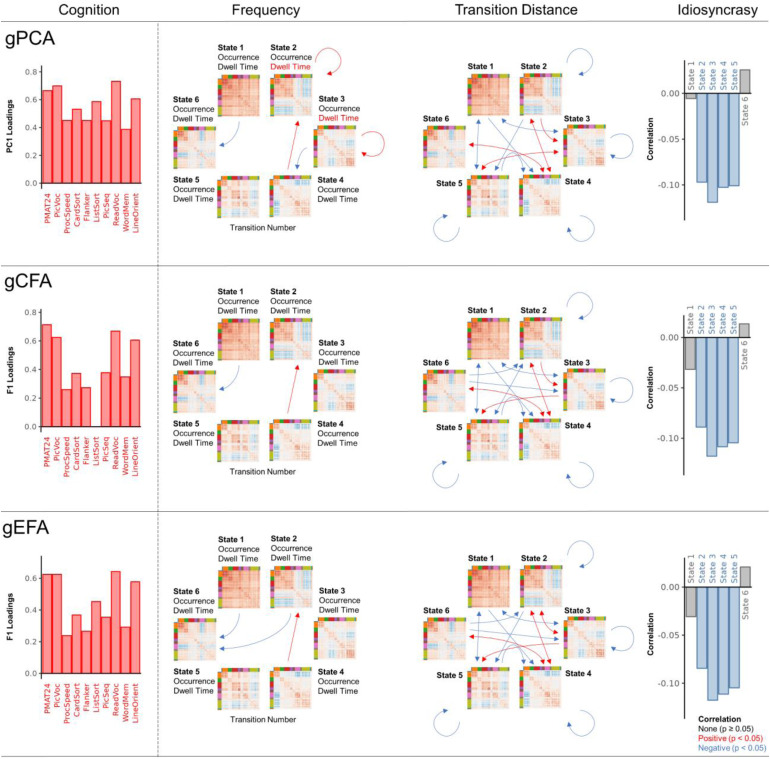
Relationship between network reconfiguration metrics and psychometric g. “gPCA” refers to the first principal component from PCA, “gCFA” refers to the g-factor from CFA, and “gEFA” refers to the g-factor from EFA. Cognitive variable loadings are shown on the left. Univariate Spearman’s correlation tests between each network reconfiguration measure and g estimate were conducted with FDR correction for 91 corresponding tests. Correlation results are shown on the right for each g estimate on the same row. Red refers to significant (FDR-corrected*p*< .05) positive correlations, blue refers to significant negative correlations. Univariate results generally replicated main findings from Dimension 1 of the PLSC. gPCA loadings, gCFA loadings, gEFA loadings, Spearman’s correlation values,*p*-values, and FDR-corrected*p*-values are located inSupplemental Table 4.

We compared Dimension 1 and g estimated solely from psychometric tests using PCA and factor analysis (seeAppendix Jfor details). Mass-univariate analyses demonstrated largely consistent results with PLSC for transition distance and idiosyncrasy, while only PCA exhibited consistent results for frequency (Fig. J.1).

#### 3.4.2 Dimension 2 reflects relationships between processing speed and network reconfiguration

In Dimension 2, the processing speed test (ProcSpeed; BR = 3.01) had the only stable loading (|BR| >2.5) and primarily loaded in the same direction as tests influenced by reaction time (Fig. 3). Dimension 2 may represent general processing speed.

While PLSC separates Dimensions 1 and 2, which we interpret as representing g and processing speed, respectively, it does not imply that g and processing speed are uncorrelated. Extensive literature characterizes a positive correlation between g and processing speed (Sheppard & Vernon, 2008). It is important to note that PLSC does not enforce orthogonality between the cognition latent variables. In fact, there is a moderate Spearman’s correlation of .27 between the cognition latent variables, supporting our interpretation. After constructing the latent variable pair with the highest covariance for Dimension 1, PLSC only ensures that the Dimension 2 cognitive latent variable is orthogonal to the Dimension 1 reconfiguration latent variable, and the Dimension 2 reconfiguration latent variable is orthogonal to the Dimension 1 cognition latent variable.

##### 3.4.2.1 Frequency relates to processing speed in Dimension 2

For Dimension 2, positive loadings were stable (|BR| >2.5) for transition number (BR = 3.51); occurrence, dwell time, within-state transition probability, and target transition probability of States 2 through 5 (occur 2, occur 3, occur 4, occur 5, dwell 3, 3-3, 5-5, 4-5; BR = 3.07, 3.47, 2.78, 3.66, 3.18, 3.29, 2.56, 2.82); and exit transition probabilities (transition probability away from the state) from States 1 and 6 (1-3, 1-4, 1-5, 6-3, 6-5; BR = 3.65, 3.02, 4.02, 2.60, 2.94) (Fig. 3). Conversely, negative loadings were stable for occurrence, dwell time, within-state transition probability, and target transition probability of State 1 (occur 1, dwell 1, 1-1, 2-1, 3-1, 4-1, 5-1, 6-1; BR = -4.25, -4.28, -3.79, -4.47, -3.54, -3.71, -3.23, -3.82). In other words, flexibility (frequent classification to different states) indexed by higher state-switching frequency, higher frequencies of States 2 through 5, and lower frequencies of States 1, primarily, and 6.

Interestingly, we observed a greater number of stable frequency-based metric loadings in Dimension 2 and a greater number of stable distance-based metric loadings in Dimension 1, suggesting broad differences in how frequency-based and distance-based metrics relate to cognition.

##### 3.4.2.2 Transition distance relates to processing speed in Dimension 2

In Dimension 2, positive loadings were stable (|BR| >2.5) for transition distances within state in all states except States 3 and 5 (1-1, 2-2, 4-4, 6-6; BR = 4.41, 2.55, 2.77, 2.59, 2.69) and between State 6 and States 1 and 2 (1-6, 6-2; BR = 2.68, 2.69) (Fig. 3) which have low transition distances across individuals (Fig. 4), indicating similar states. This implies flexibility (sequential deviation), with larger transition distances for transitions between similar states, including within state.

##### 3.4.2.3 Idiosyncrasy relates to processing speed in Dimension 2

For Dimension 2, positive idiosyncrasy loadings were stable (|BR| >2.5) for States 1 and 6 (idiosyncrasy 1, 6; BR = 4.96, 3.57) (Fig. 3). This implies atypicality and flexibility (deviation from state center) in States 1 and 6.

##### 3.4.2.4 Instability in States 1 and 6 relates to greater frequencies of States 2 through 5

**Fig. H.2.**
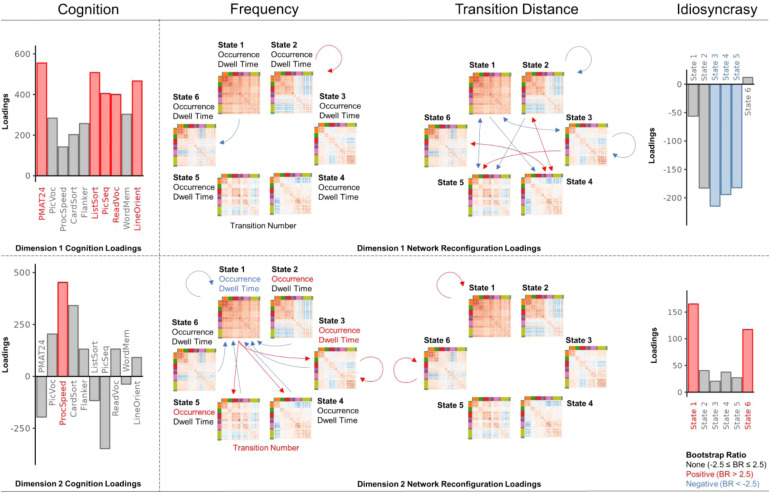
PLSC relationships between cognition and network reconfiguration metrics for |BR| >3. The same plot as the main analysis with |BR| >2.5 was done with |BR| >3.

Processing speed was linked to greater frequencies in States 2 through 5, lower frequencies in States 1 and 6, and instability in States 1 and 6, as indexed by idiosyncrasy and within-state transition distance. While processing speed was also linked to within-state transition distance in other states, delineation of States 1 and 6 by frequency and idiosyncrasy metrics suggests particular importance. Supporting this, when applying |BR| >3 (Fig. H.2), only within-state transition distances in States 1 and 6 remain. We propose that processing speed relates to instability particularly in States 1 and 6, contributing to their lower frequencies and higher frequencies in other states.

To investigate this, we also examined whether these characteristics coexist using correlations between summary measures (details inAppendix I). Spearman’s correlations were high and significant (FDR-corrected*p*< 1 × 10^-17^) (Table I.2), supporting that individuals with high idiosyncrasy and within-state transition distances in States 1 and 6 tend to have low frequencies in these states and high frequencies in States 2 through 5.

#### 3.4.3 Main findings replicate across k

We compared PLSC across*k*= 2-12. Significant (*p*< .05) and reproducible (*Z*> 1.95) Dimension 1 cognition loadings resembled g across*k*= 5-12, and significant and reproducible Dimension 2 cognition loadings resembled processing speed across*k*= 6-10 (Table B.1). Specifically, while Dimensions 1 and 2 for*k*= 2-4 were significant, neither were reproducible. For Dimension 1 (Table B.2),*k*= 6-11 exhibited higher frequencies of States 2 and 3, and lower frequencies of State 6.*k*= 5-10 exhibited efficient reconfigurations.*k*= 5-12 exhibited lower idiosyncrasy of states other than States 1 and 6. For Dimension 2 (Table B.3),*k*= 6-10 exhibited lower frequencies of States 1 and 6, higher frequencies of other states, and higher transition number.*k*= 6-10 exhibited higher transition distance in within-state transitions, including State 1 (*k*= 6, 7, 9 also included the State 6), and other low distance transitions.*k*= 6-10 exhibited higher idiosyncrasy of States 1 and 6. This suggests that our main findings replicate across ranges of*k*.

#### 3.4.4 PLSC conducted on traditional frequency-based metrics alone does not reproduce PLSC conducted on all reconfiguration metrics

We conducted PLSC using only frequency-based and distance-based metrics to examine the impact of excluding distance-based metrics on PLSC results, given high correlations between several measures (Appendix K). We found that frequency-based metrics alone do not yield identical outputs. Interestingly, the first dimension for frequency-based metrics resembles the original PLSC Dimension 2, while the first dimension for distance-based metrics resembles Dimension 1. This suggests that the strongest covariance between frequency-based metrics and cognition is the relationship with processing speed, and the strongest covariance between distance-based metrics and cognition is the relationship with g, supporting our findings of a separation in the original PLSC.

## 4 Discussion

Using PLSC, this study reveals an association between g and efficient reconfiguration between stable and typical connectivity patterns. Our findings confirm previous suggestions (Girn et al., 2019;Nomi et al., 2017) that state frequency relates to g. Specifically, g was associated with stable state maintenance of select states. We also introduced transition distance as a supplemental measure to capture individual variability in reconfiguration magnitude during state transitions. Our results indicate that g was associated with efficient transitions, with lower distances for nearby states and higher distances for distant states. We further explored a novel supplemental measure to capture interindividual idiosyncrasy, and found that g was associated with having connectivity patterns closer to the group average in specific states. Given that g was indexed by the first PLSC dimension, these data-driven associations may represent the strongest relationships between cognition and network reconfiguration. Interestingly, the second PLSC dimension related processing speed to frequent state change, higher transition distance, and greater deviation from the group average. While stability may be favorable for g, flexibility may be favorable for processing speed.

Our findings suggest a positive association between g and stability in States 2 through 5, and particularly in States 2 and 3. Higher g was related to lower within-state transition distance (sequential similarity) in States 2, 3, and 5; idiosyncrasy (similarity to state center) in States 2 through 5; and in States 2 and 3, also frequency metrics characterizing maintenance (e.g., dwell time). Within-state transitions were most frequent, so this may account for observed associations between general cognitive performance and less overall reconfiguration (Cabral et al., 2017;Hilger et al., 2020). This may represent controlled reconfiguration, consistently reaching specific connectivity patterns for g.

Importantly, frequency metrics did not implicate both States 3 and 4, which exhibited the lowest FC strength and corresponding highest FC variability. This challenges the hypothesis that these states are most relevant to g (Girn et al., 2019;Nomi et al., 2017). We propose alternative explanations. States 2 and 3 both exhibit CON-DAN coherence, CON and DAN anticoherence with DMN, and FPN-DMN coherence. CON and DAN are attentional ICNs activated during externally oriented tasks that demonstrate anticorrelation in sFC with the internally oriented DMN (Zhou et al., 2018). Intelligence has been associated with CON-DAN correlation and DAN-DMN anticorrelation (L. J. Hearne et al., 2016), suggesting that better external–internal segregation confers benefits. FPN is also externally oriented, but FPN-DMN correlation increases with intelligence and task complexity (L. Hearne et al., 2015;L. J. Hearne et al., 2016). This might relate to FPN’s task-general control function and DMN’s role in global information integration (Vatansever et al., 2015), where DMN contributes to internal idea generation, and FPN regulates DMN activity toward external goals (Beaty et al., 2018). Given that sFC can be considered a proportion-weighted sum of dFC(*t*) (Cabral et al., 2017), greater frequencies of States 2 and 3 might enhance g by amplifying beneficial communication patterns discovered from sFC. Conversely, transition probability to State 6 was inversely associated with g. State 6 was characterized by global anticoherence of FPN, suggesting isolated within-network communication. FPN modulates connectivity with other ICNs for cognitive control (Cole et al., 2013), so higher prevalence of State 6 may indicate a predisposition to enter a state where FPN does not conduct this task-general function, decreasing g. Our study underscores the importance of brain-wide stability and provides converging evidence with studies (Fraenz et al., 2021;Hilger et al., 2017,^2022^;Van Den Heuvel et al., 2009) and theories (PFIT, MD) (Duncan, 2010;Jung & Haier, 2007) for the importance of FPN, CON, DMN, and DAN to g from a dynamic perspective.

Our results demonstrated a positive association between g and the capacity to make efficient transitions. Besides stability characterized by smaller transition distances for common transitions between the same state or similar states such as States 2 and 5, higher transition distance in rarer distant transitions was also associated with higher g. This observation aligns with a finding byRamirez-Mahaluf et al. (2020), suggesting that general cognitive performance is associated with higher distance across between-state transitions. This may represent brain efficiency, where individuals with higher g make shorter or farther transitions as necessary.

Transition distance may also characterize another form of efficiency. State 1, characterized by uniform phase direction and highest frequency and target transition probability, has been interpreted as a meta-stable state that individuals tend to return to after engaging “cognitive-processing” states (Cabral et al., 2017;Vohryzek et al., 2020). Lower transition distance between State 1 and other states was associated with higher g. Prior work demonstrates that update efficiency, less required reconfiguration between resting state and task states, relates to higher cognitive performance (Schultz & Cole, 2016;Thiele et al., 2022;Xiang et al., 2022). Our findings may similarly represent efficiency, where less required reconfiguration between State 1 and cognitive-processing states relates to higher g. Together, our findings reveal novel forms of neural efficiency, extending the neural efficiency hypothesis to dynamic analysis.

Our findings suggest that higher g was associated with lower idiosyncrasy in States 2 through 5. Using a novel index of typicality, we add to the emerging view that human group averages represent an ideal by establishing a relationship between brain pattern similarity to the group average in dynamic brain states and higher cognitive performance (Corriveau et al., 2023;Gallucci et al., 2022;Hahamy et al., 2015;Hawco et al., 2020).

Our results also reveal positive associations between processing speed and general flexibility, including higher total state-switching frequency and transition distance during frequent transitions. Prior studies similarly relate processing speed to increased state switching and dFC(*t*) distance (de Lacy et al., 2019;Lombardo et al., 2020). Interestingly, processing speed was associated with higher idiosyncrasy for States 1 and 6, suggesting that atypicality in specific situations may also benefit performance. Idiosyncrasy also measures instability (deviation from state center), so this may also suggest an association between processing speed and instability in these states. This is supported by associations with within-state transition distance (sequential deviation). Importantly, processing speed was also related to reduced frequencies of States 1 and 6 and increased frequencies of States 2 through 5. We propose that, beyond general flexibility, instability in meta- and FPN-isolated states may be particularly important to processing speed by facilitating exits to beneficial cognitive-processing states.

Putting it all together, we explain the relationship between resting-state network reconfiguration and task performance, the expression of cognition, by conceptualizing resting state as an exploration of states where task demands constrain the repertoire (Deco & Jirsa, 2012). Resting state may index, for g, the capacity to efficiently achieve and sustain ideal connectivity patterns characterized by group averages in beneficial cognitive-processing states, and for processing speed, the capacity to flexibly transition between connectivity patterns in beneficial cognitive-processing states. Better escape capacity from other state types may be favorable. We predict that these capacities are expressed during tasks, but specific states hold task-specific relevance. These speculations offer avenues of exploration, particularly regarding rest–task relationships.

Beyond the specific associations for g and processing speed, we observed more stable associations between g and distance-based dFC metrics, and between processing speed and frequency-based dFC metrics. In line with this, in separate PLSC analyses of distance-based and frequency-based dFC metrics, the first dimension, explaining greatest covariance, also appeared to represent g and processing speed, respectively. This suggests broad differences in how the magnitude and frequencies of state change relate to g and processing speed. However, it is important to note that although we interpret g and processing speed separately, they are not uncorrelated. Our findings align with literature characterizing a positive correlation between g and processing speed (Sheppard & Vernon, 2008), demonstrating a moderate positive correlation between Dimensions 1 and 2 cognitive latent variables. Delving deeper into the interplay between g, processing speed, and reconfiguration represents a promising future direction.

## 5 Limitations

(1) The HCP dataset employed uses multiband fMRI. Multiband fMRI is subject to an artifact where slices simultaneously collected have shared signals (Tubiolo et al., 2023). While ICA-FIX was utilized to remove structured noise from the data, it has been shown to not fully remove the artifact. (2) While it is conventional to apply bandpass filtering before Hilbert transforms (Glerean et al., 2012), we chose not to because ICA-FIX noise removal was applied, broadband frequencies are important (Chen & Glover, 2015;Gohel & Biswal, 2015), reliability increases (Vohryzek et al., 2020), and meaningful results are found without filtering (Vohryzek et al., 2020;Wong et al., 2021). (3) Although it is common to examine*k*= 2-20 (Cabral et al., 2017), computational limitations hindered clustering at higher*k*. We deemed*k*= 2-12 sufficient to select*k*and assess robustness. We selected*k*= 6 for State 6, and confirmed its importance to g.*k*= 2-12 supported our main findings. (4) dFC patterns detected by LEiDA depend on chosen spatiotemporal scales (Cabral et al., 2017). While LEiDA is defined on single time points, the BOLD signal is relatively slow compared with neuronal firing. Moreover, our parcellation is limited to 360 cortical areas. (5) Age and gender were addressed as confounds to preserve sample size and generalizability. While we conducted extensive supplementary analyses to ensure robustness, future studies should more deeply explore the impact of multiband fMRI artifact correction, bandpass filtering,*k*, other spatiotemporal scales (e.g., magnetoencephalography, subcortical, voxel wise), and age and gender differences using broader samples, such as HCP Lifespan Studies (Bookheimer et al., 2019) or NKI Rockland Sample (Nooner et al., 2012).

## 6 Future Directions

Although whole-brain metrics demonstrated statistically significant associations with g, magnitudes were moderate, precluding that these metrics fully account for g. Integrating multiscale findings may offer additional insight (Barbey, 2018;Girn et al., 2019). For instance, higher regional dFC(*t*) flexibility, rather than stability, is linked to enhanced performance across cognitive abilities (Bassett et al., 2011;Braun et al., 2015;Jia et al., 2014), suggesting that increased flexibility in regions, albeit not enough to affect brain-wide pattern stability, may benefit g. Likewise, sFC connections that predict behavior exhibit greater consistency across participants than nonpredictive connections, but the most predictive connections among predictive connections are the least consistent (Greene et al., 2020). Having more idiosyncrasy in connections, but not enough to affect brain-wide typicality, may also benefit g.

The goal of this research is cognitive enhancement in impaired, or even healthy, individuals. Unlike existing approaches with transient effects or issues such as impaired plasticity (Schifano et al., 2022), effective enhancement requires comprehensive understanding through studies such as the present analysis. This understanding can also have other applications. Conceptually related to our findings implicating dynamic efficient control of optimal connectivity patterns in facilitating g, brain connectivity might be purposefully dynamic and designed to minimize energy expended in transitioning between target brain activation patterns (Deng et al., 2022). Another application may be to implement dynamic, efficient, controlled, and optimal adaptation of network weights akin to the brain to improve neural network performance.

## 7 Conclusions

In summary, our framework (1) finds that g is associated with controlled and efficient transitions between stable and typical connectivity states, (2) suggests that processing speed is associated with flexible connectivity, and (3) emphasizes the importance of examining connectivity in more individualized ways than just state frequency. Our findings shed light on novel and important principles governing brain organization and information processing, paving the way toward cognitive enhancement and other innovative applications.

## Supplementary Material

Supplementary Material

Supplementary Table 1

Supplementary Table 2

Supplementary Table 3

Supplementary Table 4

## Figures and Tables

**Fig. A.1.**
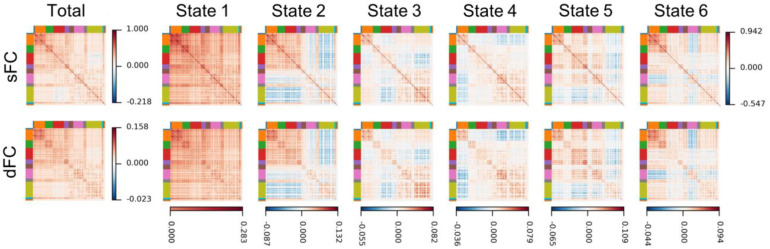
Correspondence between phase dFC and sFC. The matrix denoted by Total and dFC refers to the state occurrence-weighted sum of phase dFC patterns. The matrices denoted by State # and dFC refer to the average phase dFC state patterns. The matrix denoted by Total and sFC refers to Pearson’s correlations between BOLD signals from all timepoints. The matrices denoted by State # and sFC refer to Pearson’s correlations between BOLD signals from all timepoints classified to the state.

**Fig. B.2.**
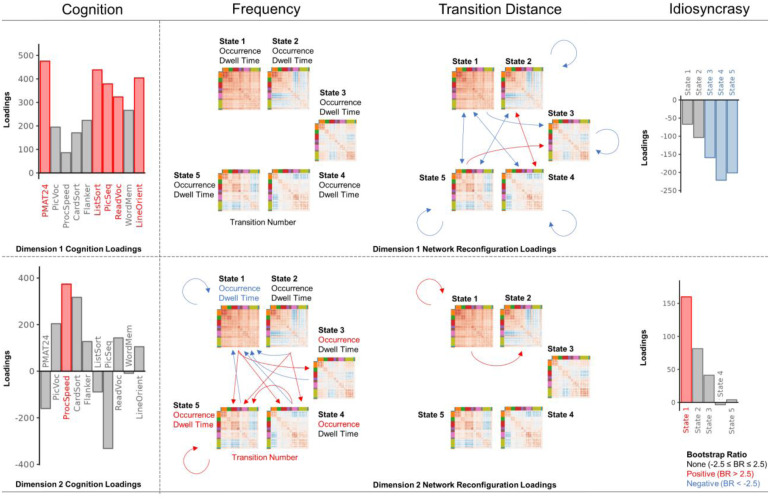
PLSC relationships between cognition and network reconfiguration metrics for*k*= 5. Red refers to stable positive bootstrap ratios (|BR| >2.5) for original variable loadings on the latent variables, blue refers to stable negative bootstrap ratios. Arrows are transition probabilities for frequency metrics and transition distances for transition distance; circular arrows represent within-state transitions or distances. Each dimension refers to a single pair of cognition and network reconfiguration latent variables. For the Dimension 1 cognitive test loadings, most loadings were positive—this suggests that it represents g. For the Dimension 2 cognitive test loadings, only processing speed was stable. Interestingly, an even more extreme division of stable transition distance loadings to the first PLSC dimension, and frequency metric loadings to the second PLSC dimension is observed compared with*k*= 6, reinforcing the observation of differences in how the frequencies and magnitudes of state change relate to g and processing speed.

**Fig. D.1.**
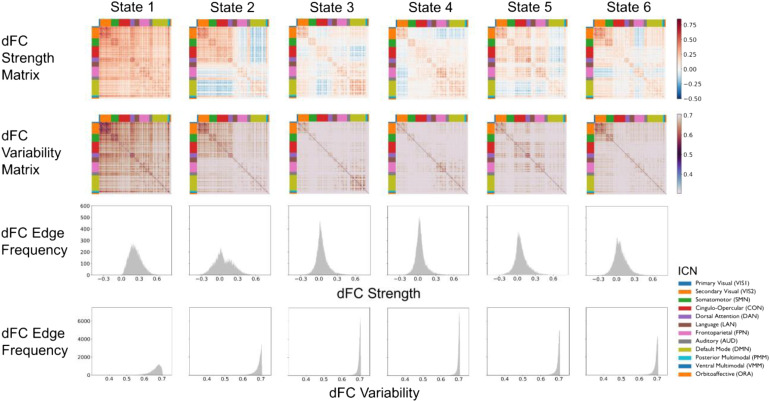
dFC strength and variability plots. In the first and second rows, FC strength and FC variability in each connection are represented in matrices. Red colors refer to positive coherence, white colors refer to zero coherence, and blue colors refer to negative coherence for FC strength. Darker colors refer to lower FC variability for FC variability. In the third and fourth rows, histograms for the frequency of connections at each value for the matrix representations characterizing FC strength and FC variability are plotted.

**Fig. E.1.**
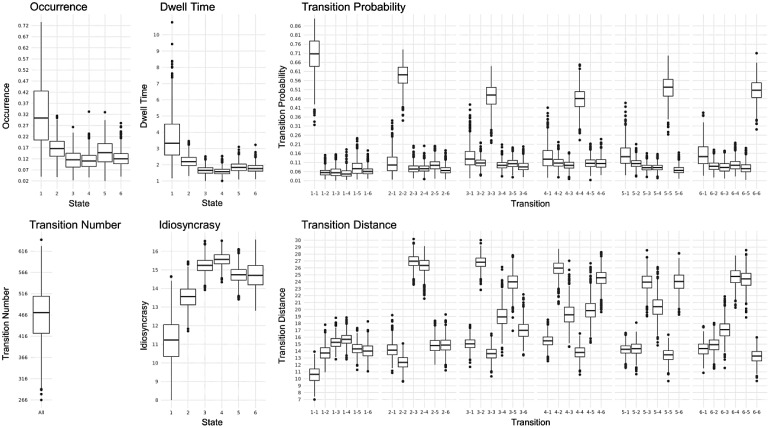
Box plots for the individual distributions of network reconfiguration metrics. For each box plot, x-axis labels are the network reconfiguration metrics and y-axis values quantify the distributions of values across individuals.

**Fig. F.1.**
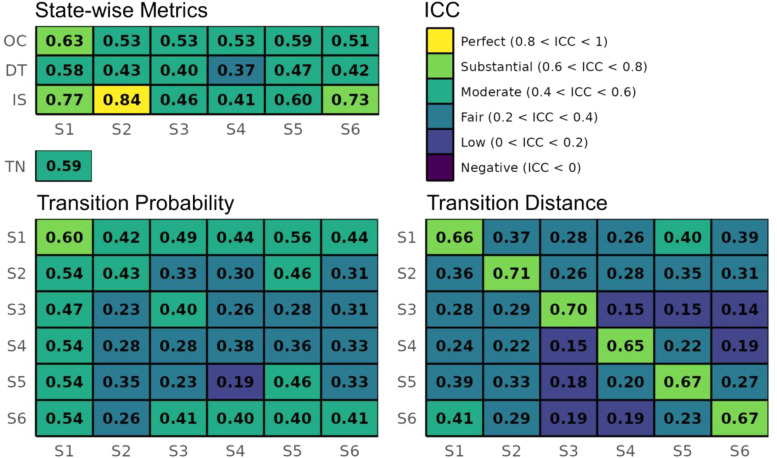
Network reconfiguration ICC. For the state-wise metrics, OC refers to occurrence, DT refers to dwell time, and IS refers to idiosyncrasy. TN refers to the transition number. For the transition probability and transition distance plots, S# refers to the state. The y-axis denotes the starting state and the x-axis denotes the ending state. Each cell is colored by the ICC interpretation (Landis & Koch, 1977).

**Fig. G.1.**
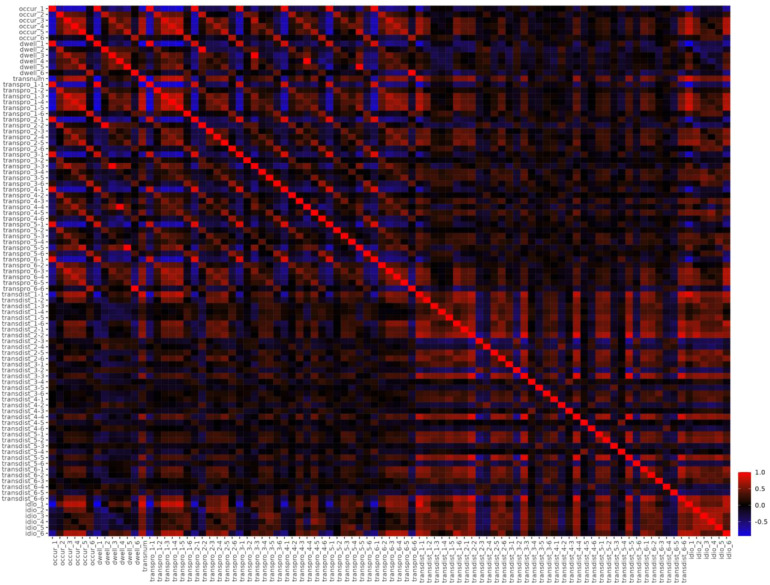
Correlations between reconfiguration metrics. We found Spearman’s correlations for each variable. Red indicates positive values, blue indicates negative values. Reconfiguration metrics are organized from frequency, to transition distance, to idiosyncrasy metrics. “Occur” refers to occurrence, “dwell” refers to dwell time, “transnum” refers to transition number, “transpro” refers to transition probability, “transdist” refers to transition distance, and “idio” refers to idiosyncrasy. Correlation values are located inSupplemental Table 1.

**Fig. G.2.**
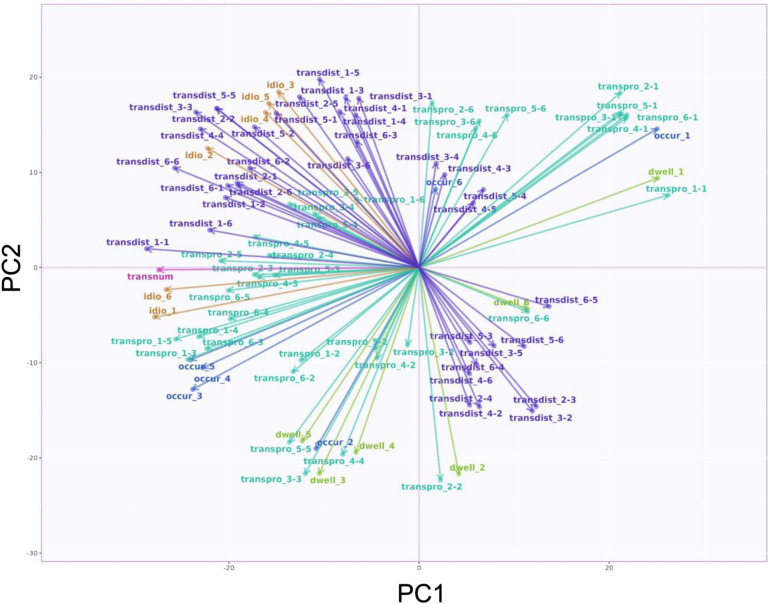
PCA plot of reconfiguration metric loadings. We plotted the loadings of reconfiguration metrics on PC1 and PC2 from a PCA of all reconfiguration metrics as arrows. A 0° angle indicates perfect positive correlation, a 180° angle indicates perfect negative correlation, and a 90° angle indicates orthogonality. “Occur” refers to occurrence, “dwell” refers to dwell time, “transnum” refers to transition number, “transpro” refers to transition probability, “transdist” refers to transition distance, and “idio” refers to idiosyncrasy. Occurrence, dwell time, transition number, transition probability, transition distance, and idiosyncrasy metrics are color coded.

**Fig. K.1.**
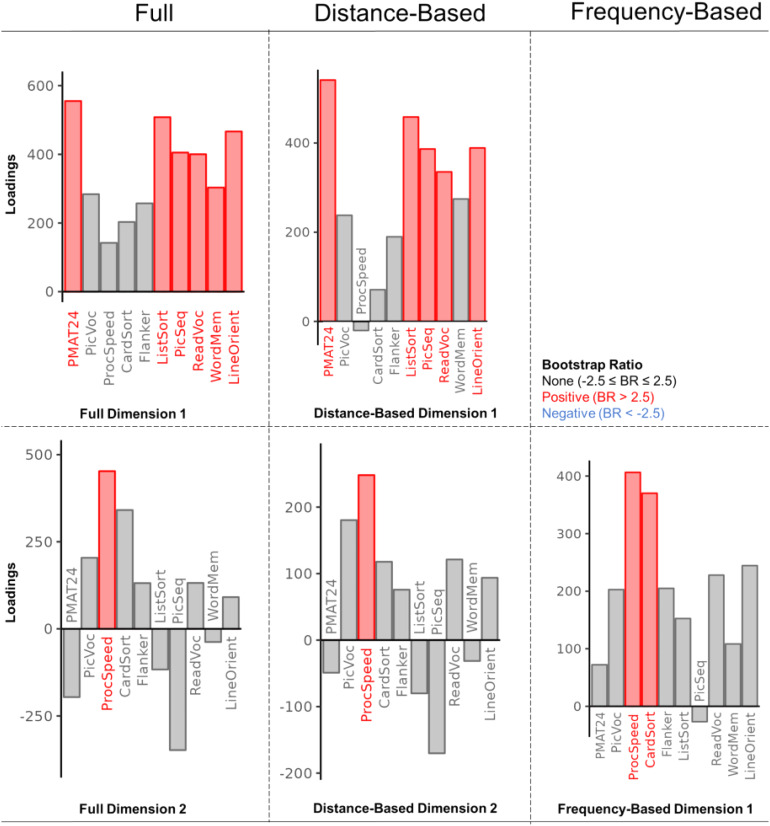
PLSC comparison for frequency-based and distance-based reconfiguration metrics. The left two bar plots represent the loadings for Dimensions 1 and 2 of the original PLSC. We have repeated PLSC on the frequency-based and distance-based reconfiguration metrics alone. The middle two bar plots represent the loadings for Dimensions 1 and 2 of the distance-based PLSC, and the right bottom bar plot represents the loadings for Dimension 1 of the frequency-based PLSC.

## Data Availability

The HCP1200 dataset (S. M. Smith et al., 2013) can be accessed at:https://db.humanconnectome.org/. All relevant code for this analysis can be found at:https://github.com/14jwkn/FLEXCOG.

## Appendix

### Appendix A. d FC- s FC Correspondence

Similar to prior work (Cabral et al., 2017), we observed that sFC and the sum of state occurrence-weighted phase dFC matrices across time were nearly identical in pattern with a Spearman’s correlation of .988 (Fig. A.1). In addition, extracting timepoints classified to each phase dFC state and calculating Pearson’s correlations between the timepoints also generate connectivity matrices very similar to the average phase dFC pattern in each state, with a Spearman’s correlation of .993 for State 1, .999 for State 2, .998 for State 3, .996 for State 4, .997 for State 5, and .999 for State 6.

### Appendix B. *k* = 2-12

We repeated*k*-medians clustering for*k*= 2-12 to choose*k*for the main analysis and to investigate whether our main findings replicate across*k*post hoc. InFigure B.1, we show LE matrices and brain plots which we used to select*k*= 6 for the main analysis. InTable B.1, we show whether there are also significant (*p*< .05) and reproducible (*Z*> 1.95) dimensions which produce cognition latent variable loadings which resemble g (i.e., all tests positive) and processing speed (i.e., ProcSpeed stable, tests including reaction time such as CardSort and Flanker positive) as in the main analysis. InTable B.2, we show, for significant and reproducible dimensions which resemble g, whether main findings replicate. InTable B.3, we show, for significant and reproducible dimensions which resemble processing speed, whether main findings replicate. We also replicateFigure 3with*k*= 5 inFigure B.2as an example with a k commonly used across studies (Cabral et al., 2017;Damaraju et al., 2014;Nomi et al., 2017).

**Table B.1.** PLSC diagnostics and cognition latent variables across*k*= 2-12.

	Significant	Reproducible	LVY1 resembles g	LVY2 resembles processing speed
*k* = 2	D1, D2	No	—	—
*k* = 3	D1, D2	No	—	—
*k* = 4	D1, D2	No	—	—
*k* = 5	D1, D2, D3	D1	Yes	—
*k* = 6	D1, D2, D3	D1, D2	Yes	Yes
*k* = 7	D1, D2, D3	D1, D2	Yes	Yes
*k* = 8	D1, D2, D3	D1, D2	Yes	Yes
*k* = 9	D1, D2, D3	D1, D2	Yes	Yes
*k* = 10	D1, D2, D3	D1, D2	Yes	Yes
*k* = 11	D1, D2, D3	D1	Except ProcSpeed	—
*k* = 12	D1, D2, D3	D1	Yes	—

Each row corresponds to one*k*. In the “Significant” column, the dimensions (D) which were significant (*p*< .05) based on permutation testing are listed. In the “Reproducible” column, the listed dimensions (D) have SV, latent variables for**Y**(LVY) cognition variables, and latent variables for**X**(LVX) network reconfiguration variables which all have reproducible scores (*Z*> 1.95) based on split-half analysis. “LVY1 resembles g” column states whether Dimension 1 contains a**Y**cognition latent variable (LVY1) with loadings which are all positive and thus resembles g. “LVY2 resembles processing speed” column states whether Dimension 2 contains a**Y**cognition latent variable (LVY2) with loadings for ProcSpeed which are stable (|BR| >2.5) and positive for tests directly including reaction time in the calculation including CardSort and Flanker. Across*k*= 2-12, no more than three dimensions exhibited both significance and reproducibility. For*k*= 5-12, Dimension 1 displayed significance, reproducibility, and loadings resembling g. For*k*= 6-10, Dimension 2 displayed significance, reproducibility, and loadings resembling processing speed.

**Table B.2.** Main findings for g across*k*= 5-12.

	+State 2, +State 3, -State 6	Efficient	Typicality besides States 1 & 6
*k* = 5	No	Yes	Yes
*k* = 6	**State 2:** Maintenance (+), Target transition (+) **State 3:** Maintenance (+) **State 6:** Target transition (-)	Yes	Yes
*k* = 7	**State 2:** Maintenance (+), Target transition (+), Exit transition (-) **State 6:** Target transition (-)	Yes	Yes
*k* = 8	**State 2:** Maintenance (+), Exit transition (-) **State 3:** Exit transition (-) **State 6:** Target transition (-)	Yes	Yes
*k* = 9	**State 2:** Maintenance (+), Target transition (+), Exit transition (-) **State 6:** Target transition (-)	Yes	Yes
*k* = 10	**State 2:** Maintenance (+), Target transition (+), Exit transition (-) **State 6:** Target transition (-)	Yes	Yes
*k* = 11	**State 2:** Target transition (+) **State 3:** Exit transition (-) **State 6:** Target transition (-)	No	Yes
k = 12	No	No	Yes

Each row corresponds to one*k*.*k*where Dimension 1 is significant (*p*< .05) and contains reproducible (*Z*> 1.95) SV, latent variable loadings for**Y**(LVY) cognition variables, and latent variable loadings for**X**(LVX) network reconfiguration variables, and resembles g (all tests positive) are examined. Stable (|BR| >2.5) network reconfiguration loadings are interpreted. (+) refers to positive relationships with g, (-) refers to negative relationships. Column 1 records whether higher g related to greater frequency of States 2 and 3 and lower frequency of State 6. Higher frequency is recorded as higher maintenance (dwell time, within-state transition probability), higher target transition probability, or lower exit transition probability. The reverse is also true. Column 2 records whether higher g related to higher transition distances among distant transitions and lower transition distances among short transitions. Column 3 records whether higher g related to lower idiosyncrasy of states other than States 1 and 6. For*k*= 6-11, higher g was associated with higher frequency of States 2 and 3, and lower frequency of State 6. For*k*= 5-10, higher g was associated with greater transition distance for transitions between dissimilar states and lower transition distance for transitions between similar states. For*k*= 5-12, higher g was associated with lower idiosyncrasy of states other than States 1 and 6.

**Table B.3.** Main findings for processing speed across*k*= 6-10.

	-State 1, -State 6, +State N, +Transition Number	Flexible	Idiosyncrasy of States 1 & 6
*k* = 6	**State 1:** Exit transition (+), Prevalence (-), Target transition (-) **State 6:** Exit transition (+) **State N:** Prevalence (+), Target transition (+) **Transition Number:** Yes	Yes, including States 1 & 6	Yes
*k* = 7	**State 1:** Exit transition (+), Prevalence (-), Target transition (-) **State 6:** Exit transition (+) **State N:** Frequency (+), Target transition (+) **Transition Number:** Yes	Yes, including States 1 & 6	Yes
*k* = 8	**State 1:** Exit transition (+), Prevalence (-), Target transition (-) **State 6:** Exit transition (+) **State N:** Prevalence (+), Target transition (+) **Transition Number:** Yes	Yes, including State 1	Yes
*k* = 9	**State 1:** Exit transition (+), Prevalence (-), Target transition (-) **State 6:** Exit transition (+) **State N:** Prevalence (+), Target transition (+) **Transition Number:** Yes	Yes, including States 1 & 6	Yes
*k* = 10	**State 1:** Exit transition (+), Prevalence (-), Target transition (-) **State 6:** Exit transition (+) **State N:** Prevalence (+), Target transition (+) **Transition Number:** Yes	Yes, including State 1	Yes

Each row corresponds to one*k*.*k*where Dimension 2 is significant (*p*< .05) and has reproducible (*Z*> 1.95) SV, latent variable loadings for**Y**(LVY) cognition variables, and latent variable loadings for**X**(LVX) network reconfiguration variables, and resembles processing speed (ProcSpeed stable, CardSort, and Flanker positive) are examined. Stable (|BR| >2.5) network reconfiguration loadings are interpreted. (+) refers to positive relationships with processing speed, (-) refers to negative relationships. Column 1 records whether higher processing speed related to lower frequencies of States 1 and 6, higher frequency of other N states, and higher transition number. Higher frequency is recorded as higher prevalence (occurrence, dwell time, within-state transition probability), higher target transition probability, and lower exit transition probability. The reverse is also true. Column 2 records whether higher processing speed related to higher transition distance in within-state and other low distance transitions, and whether this included within-state transition distance of States 1 and 6. Column 3 records whether higher processing speed was related to higher idiosyncrasy of States 1 and 6. For*k*= 6-10, higher processing speed was associated with lower frequencies of States 1 and 6, higher frequencies of other N states, and higher transition number. For*k*= 6-10, higher processing speed was associated with higher transition distance in within-state transitions, including State 1, and other low distance transitions. For*k*= 6, 7, and 9, State 6 within-state transition was also included. For*k*= 6-10, higher processing speed was associated with higher idiosyncrasy of States 1 and 6.

### Appendix C. Extended Methods

*2.9.1.4. Cross-validation for PLSC:*We conducted 10-fold cross-validation, randomly splitting individuals into a 90% train set and a 10% test set. We accounted for family structure by making sure that related individuals were not separated between splits (Ooi et al., 2022). We also accounted for data leakage by conducting confound regression and standardization on the test set using coefficients, mean, and standard deviation (*SD*) derived from the train set. We conducted PLSC on the train set. We multiplied the train set network reconfiguration and cognition singular vectors (i.e., weights of the linear combination) with the corresponding test set matrices to calculate estimated latent variables for the test set. We repeated this for every fold, concatenating the test set values to estimate PLSC latent variables with values for every individual in the full sample. To cross-validate the observed associations, we computed the Spearman’s correlation between the estimated reconfiguration latent variables and the estimated cognition latent variables to assess the out-of-sample association strength. To cross validate the reconfiguration latent variables and cognition latent variables separately, per se, we also computed the Spearman’s correlations between the estimated latent variables and the original PLSC latent variables to examine their consistency.

*2.9.1.5. Variance explained for PLSC:*To quantify the amount of variance in the original variables explained by the latent variables, we computed the sums of the squared Pearson’s correlations between the original and latent variables. By dividing the explained variance by the largest possible covariance PLSC can explain, the multiplication of the number of original variables and the number of latent variables, we quantify the size of the effect. To calculate the out-of-sample variance explained, we split the individuals into the train and the test sets and repeated the calculation. Original variables are extracted for the test set and latent variables are estimated for the test set using the singular vectors derived from the train set. This procedure was repeated 10,000 times for the reproducibility analysis and 10 times for the cross-validation analysis, and the variance explained was averaged across all 10,000 and 10 splits, respectively.

### Appendix D. d FC Strength and Variability

Nomi et al. (2017)observed a positive association between maintaining dFC states characterized by low FC strength (i.e., magnitude of connectivity values) and high FC variability (i.e., variability of connectivity values over time) and executive function. Building on this,Girn et al. (2019)proposed that sustaining states with high FC variability exemplifies the flexibility described by the Network Neuroscience Theory of Human Intelligence, and thus should relate to g. To address this hypothesis, we investigated FC strength and FC variability in dFC(*t*). Following the method described byNomi et al. (2017), for each connection, we calculated FC variability using the*SD*of the phase difference across time, and FC strength using the average.

To verify the correspondence between FC strength and FC variability, we concatenated dFC(*t*) across all states, and calculated FC strength and FC variability within dFC(*t*) classified to each run and individual. Then, we averaged these values across runs and individuals. We calculated the Spearman’s correlation between the absolute value of FC strength and FC variability. To identify candidate states whose maintenance should relate to g, we concatenated dFC(*t*) within each state, we repeated the same steps. We plotted the FC strength and FC variability for each connection as matrices and histograms for comparison between states to identify states with low FC strength and high FC variability (Fig. D.1). Connections with high FC strength tended to have low FC variability. States with lower FC strength across connections (near zero) tended to have the higher FC variability (far from zero), and States 3 and 4 tended to have connections with the lowest FC strength and highest FC variability.

### Appendix E. Individual Distributions of Network Reconfiguration Metrics

To characterize the network reconfiguration metrics, we show the distribution of network reconfiguration metric scores for each individual (Fig. E.1). For transitions to the same state, transition probabilities were among the highest and transition distances were among the lowest. Both directions of transition distances for each pair of states displayed similar distributions. Occurrence, dwell time, within-state transition probability, and target transition probability for State 1 were among the highest.

### Appendix F. Test–Retest Reliability of Network Reconfiguration Metrics

Test**–**retest reliability assesses the consistency of a metric across repeated tests. Complementary to our goal of investigating whether network reconfiguration metrics can predict general intelligence, we characterized the consistency of the network reconfiguration metrics across repeated tests to understand how well each metric represents a stable trait in the individual. Building upon prior research demonstrating test**–**retest reliability of frequency metrics using LEiDA (Vohryzek et al., 2020), we examined the consistency of the novel metrics of transition distance and idiosyncrasy.

We assessed the test**–**retest reliability of network reconfiguration metrics using the Intraclass Correlation Coefficient (ICC), which measures the ratio of within-individual variability to between-individual variability (Shrout & Fleiss, 1979). Specifically, we employed ICC(1,1), a standard approach in similar analyses (Noble et al., 2019). ICC is derived from the formula (MSE_b_- MSE_w_)/(MSE_b_+ MSE_w_), where MSE_w_is the within-individual mean-squared error and MSE_b_is the between-individual mean-squared error. The maximum ICC value is 1. Positive ICC values indicate that within-individual variability is lower than between-individual variability, indicating that the assessed metric can distinguish individuals from a group (Vohryzek et al., 2020). ICC values are typically categorized as low (0-.2), fair (.2-.4), moderate (.4-.6), substantial (.6-.8), and near perfect (.8-1) (Landis & Koch, 1977).

To assess ICC for each network reconfiguration metric, we averaged the metric across two phase-encoding runs (e.g., REST1_LR and REST1_RL) within each of two sessions (rather than both sessions in the main analysis), which were conducted on separate days. This approach reduces the influence of phase-encoding direction, compared with conducting pairwise comparisons between all runs.

All network reconfiguration metrics displayed positive ICC (Fig. F.1), attesting to their ability to distinguish individuals from a group (Vohryzek et al., 2020). ICC for frequency metrics was consistent with prior studies employing both LEiDA and other dFC approaches (Choe et al., 2017;Li et al., 2020;D. M. Smith et al., 2018;Vohryzek et al., 2020;Zhao et al., 2019). Most occurrence and dwell time metrics demonstrated moderate ICC, and the majority of transition probability metrics displayed only fair ICC. However, frequency metrics for State 1 demonstrated particularly high ICC. Occurrence and within-state transition probability for State 1 demonstrated substantial ICC, and other transition probability metrics involving State 1 demonstrated the highest ICC among transitions, though their ICC values were moderate.

Interestingly, we observed that idiosyncrasy and within-state transition distance ICC were particularly high, with State 2 idiosyncrasy ICC being near perfect. However, transition distance for other transitions generally exhibited fair to low ICC. Particularly low ICC was observed for transitions between State 3 and States 4 to 6. One potential explanation is the infrequency of certain transitions, which may hinder accurate estimation of ICC despite the 30-minute scan length employed in HCP.

### Appendix G. Relationships Between Reconfiguration Metrics

To assess whether both traditional frequency-based and novel distance-based reconfiguration metrics represent the same dimensions of individual variability, we assessed their correlations (Fig. G.1) and a PCA of all reconfiguration metrics (Fig. G.2). Within-modality correlations tend to be the highest. While correlations are high between idiosyncrasy and frequency metrics (upper right bright band/lower left bright band), correlations are generally much lower between transition distance metrics and frequency metrics (upper right dark block/lower left dark block). When plotting the reconfiguration loadings of the first two PCs from the PCA (explaining 45.11% of the variance) as arrows to visualize the underlying factor structure of these metrics, we observe a divergence between both transition distance/idiosyncrasy distance-based and frequency-based metrics. The relationship between any two metrics is quantified by the cosine of the angle formed between the arrows for the corresponding loadings. For instance, a 0° angle indicates perfect positive correlation, a 180° angle indicates perfect negative correlation, and a 90° angle indicates orthogonality. The distance-based metrics tend to project from the upper left quadrant to bottom right quadrant, while frequency-based metrics tend to project from the bottom left quadrant to upper right quadrant, indicating orthogonality between these two metric sets. This suggests that the variability of the distance-based metrics and the frequency-based metrics explains nonoverlapping variance of the data and assesses different aspects of network reconfiguration.

### Appendix H. Additional Outputs for *k* = 6 PLSC

We conducted PLSC by setting the 10 cognitive tests as the**Y**matrix and the 91 network reconfiguration variables as the**X**matrix. Permutation test outputs, reproducibility analysis outputs, and latent variable Spearman’s correlations are shown inFigure E.1. For the permutation tests (Fig. H.1A), the first three dimensions were significant (*p*< .05). For the reproducibility analysis (Fig. H.1B), PLSC variables for all dimensions were reproducible (*Z*> 1.95) except for the SV and the network reconfiguration latent variables for the third dimension, so we removed the third dimension from further analyses. For the latent variables correlations (Fig. H.1C), the overall correlations were moderate.

PLSC for the more stringent |BR| >3 assessed post hoc is shown inFigure H.2, in contrast to |BR| >2 in the main analysis. Except for minor differences for cognition and network reconfiguration loadings at the cusp of stability, stable loadings generally support the main findings. Of note, transition distance metrics predominantly met the higher threshold for Dimension 1, whereas frequency metrics primarily met the higher threshold for Dimension 2. This observation reinforces the notion that the frequencies and magnitudes of state change differentially relate to g and processing speed. Interestingly, within-state transition distances of States 1 and 6 were specifically retained for Dimension 2, aligning with our interpretation that higher within-state instability in these states is more important to processing speed.

### Appendix I. Correlations Between Summary Measures of Network Reconfiguration

We examined whether network reconfiguration characteristics associated with g covary post hoc. By averaging metrics stably (|BR| >2.5) associated with better cognitive performance on Dimension 1 of the PLSC (seeFigs. 3and4), we generated a “maintenance” score from dwell time and within-state transition probabilities which have positive loadings (dwell 2, dwell 3, 2-2, 3-3), a “within-state” distance score from within-state transition distances which have negative loadings (2-2, 3-3, 5-5), a “between-positive” score from between-state transition distances with positive loadings which tend to be between dissimilar states (2-3, 2-4, 3-5, 4-2, 4-6, 6-4), a “between-negative” score from between-state transition distances with negative loadings which tend to be between similar states (1-3, 1-4, 1-5, 2-5, 3-1, 5-1), and an “idiosyncrasy” score from idiosyncrasy scores which have negative loadings (idiosyncrasy 2, 3, 4, 5).

We also examined whether network reconfiguration characteristics associated with processing speed covary. By averaging metrics stably (|BR| >2.5) associated with better cognitive performance on Dimension 2 of the PLSC (seeFigs. 3and4), we generated the scores. Importantly, we did not use the same metrics which may apply in two scores to avoid circularity. “2-3-4-5” was generated from frequency metrics characterizing higher frequencies of States 2 through 5 (i.e., higher occurrence, higher dwell time, higher within-state transition probability, and higher target transition probability) which have positive loadings (occur 2, occur 3, occur 4, occur 5, dwell 3, 3-3, 5-5, 4-5), “Less 1” was generated from frequency metrics characterizing lower frequency of State 1 (i.e., higher exit transition probability) which have positive loadings (1-3, 1-4, 1-5), “More 1” was generated from frequency metrics characterizing higher frequency of State 1 (i.e., lower occurrence, lower dwell time, lower within-state transition probability, lower target transition probability) which have negative loadings (occur 1, dwell 1, 1-1, 2-1, 3-1, 4-1, 5-1, 6-1), “Less 6” was generated from frequency metrics characterizing lower frequency of State 6 (i.e., higher exit transition probability) which have positive loadings (6-3, 6-5), “Within 1 & 6” was generated from within-state transition distance metrics for States 1 and 6 which have positive loadings (1-1, 6-6), and “Idiosyncrasy 1 & 6” (idiosyncrasy 1, idiosyncrasy 6) was generated from idiosyncrasy metrics for States 1 and 6 which have positive loadings.

We conducted confound regression with age and gender (Agelink van Rentergem et al., 2020) for each variable prior to averaging, used Spearman’s correlation tests to examine pairwise relationships, and applied FDR correction across 10 tests for g and 15 tests for processing speed. The results are reported inTable I.1for g andTable I.2for processing speed. All correlations were significant (FDR-corrected*p*< 1 × 10^-17^) and generally high, indicating that network reconfiguration characteristics covary.

**Table I.1.** Correlations between summary reconfiguration metrics related to g.

	Maintenance (+)	Within (-)	Between-Positive (+)	Between-Negative (-)	Idiosyncrasy (-)
**Maintenance (+)**	—-	-0.28	0.37	-0.49	-0.42
**Within (-)**	—-	—-	-0.61	0.80	0.73
**Between-Positive (+)**	—-	—-	—-	-0.57	-0.63
**Between-Negative (-)**	—-	—-	—-	—-	0.65
**Idiosyncrasy (-)**	—-	—-	—-	—-	—-

Higher (+) or lower (-) network reconfiguration values associated with higher g (all FDR-corrected*p*< 1 × 10^-17^).

**Table I.2.** Correlations between summary reconfiguration metrics related to processing speed.

	2-3-4-5 (+)	Less 1 (+)	More 1 (-)	Less 6 (+)	Within 1 & 6 (+)	Idiosyncrasy 1 & 6 (+)
**2-3-4-5 (+)**	—-	0.87	-0.91	0.74	0.55	0.79
**Less 1 (+)**	—-	—-	-0.91	0.78	0.72	0.90
**More 1 (-)**	—-	—-	—-	-0.67	-0.63	-0.81
**Less 6 (+)**	—-	—-	—-	—-	0.65	0.82
**Within 1 & 6 (+)**	—-	—-	—-	—-	—-	0.85
**Idiosyncrasy 1 & 6 (+)**	—-	—-	—-	—-	—-	—-

Higher (+) or lower (-) network reconfiguration values associated with higher processing speed (all FDR-corrected*p*< 1 × 10^-17^).

For g-related characteristics, lower within-state transition distance was associated with higher between-state transition distance for transitions between dissimilar states, lower between-state transition distance for transitions between similar states, and lower idiosyncrasy.

For processing speed-related characteristics, higher frequencies of States 2 through 5 were associated with lower frequency of State 1, lower frequency of State 6, higher within-state transition distances of States 1 and 6, and higher idiosyncrasy of States 1 and 6. Of note, the idiosyncrasy summary score produced higher correlations with frequency scores than within-state transition distance summary scores. This may relate to how these indices of instability were calculated. While within-state transition distance was calculated as the average distance between sequential LE(*t*), idiosyncrasy was calculated by the average distance of all LE(*t*) to the state center. Perhaps the latter is a better measure of within-state instability as it relates to frequency.

### Appendix J. Psychometric g

We interpreted the cognition latent variable of the first PLSC dimension as a measure of g. However, the cognition latent variable was constructed with information from network reconfiguration metrics, which may be at odds with the definition of g as a psychometric construct. We also investigated whether our results replicate for purely psychometrically defined estimates of g post hoc.Dubois et al. (2018)fitted factor analysis models to the 10 HCP cognitive tests used in the PLSC to estimate g. Individual scores on factors run into the problem of factor score indeterminacy, where different but valid factor scores can be extracted from the same factor model (DiStefano et al., 2009). The standardized sum of test scores is a simple method which does not directly account for factor analysis loadings, butDubois et al. (2018)rationalized not using the standardized sum because this gives equal weights to all tests despite our use of two differently scaled batteries. Another simple, but weighted, method is PCA, which is similar to PLSC in that it produces orthogonal latent dimensions, but different in that PCA generates weighted latent variables which maximize the total variance retained across test scores, rather than the total covariance between test scores and brain variables as in PLSC. The first principal component, accounting for the majority of variance, is commonly used in neuroscience literature to estimate g (Cremers et al., 2019;Hoogendam et al., 2014;Trampush et al., 2017). But refined factor scoring methods which account for factor analysis loadings might better characterize g as a construct. Factor scores can be extracted from both an Exploratory Factor Analysis (EFA) which generates factors based on the unknown factor structure and a Confirmatory Factor Analysis (CFA) which tests a provided factor structure (Dubois et al., 2018). CFA is preferred to derive factor scores, but there is little difference between CFA and EFA for deriving g. Both can be reported to confirm this. To contrast with our PLSC results, we investigated gPCA derived from PCA, gCFA from CFA, and gEFA from EFA.

For gPCA, we conducted a PCA on the 10 cognitive test scores for the same sample and extracted the first principal component. For the factor analyses, we implemented procedures similar toDubois et al. (2018). In brief, first, we used an expanded sample of 1192 individuals (male = 548, age = 28.82 ± 3.69 years; race = 878 White, 192 Black, 67 Asian, 31 multiple, 22 unknown, and 2 American Indian) to improve factor estimation by only constraining the sample to those that have all 10 cognitive test scores. We conducted EFA using the psych package (Revelle, 2016) by fitting a bifactor model where g loads onto all 10 cognitive tests and several orthogonal group factors load onto subsets to account for remaining common variance across tests. In the omega function, a factor analysis with maximum-likelihood estimation is done, the factors are rotated obliquely, the correlation matrix constructed on the factors is factored, and a Schmid–Leiman transformation is done to extract factor loadings. We conducted CFA using the lavaan package (Rosseel, 2012) using the factor structure discovered in EFA, but without cross-loadings of any task onto multiple group factors. Of note, ListSort was removed because the lavaan model does not converge otherwise, perDubois et al. (2018). gEFA and gCFA factor scores were derived from the respective models using the regression method.

To contextualize the estimates of g, we show Spearman’s correlations inTable J.1between the cognition latent variable for PLSC Dimension 1 (gPLSC), gPCA, gCFA, gEFA, and the 10 original cognitive test scores. gPLSC and gPCA are more correlated, and gCFA and gEFA are more correlated, as expected from their methods of construction. However, all correlations between g estimates were greater than .9, supporting their similarity.

**Table J.1.** Correlations between estimates of g and original cognitive test scores.

	gPLSC	gPCA	gCFA	gEFA	PMAT24	PicVoc	CardSort	Flanker	ListSort	PicSeq	ProcSpeed	ReadVoc	WordMem	LineOrient
**Gplsc**	**1**	**0.97**	**0.95**	**0.95**	0.7	0.63	0.43	0.35	0.63	0.52	0.36	0.67	0.39	0.59
**gPCA**	**0.97**	**1**	**0.92**	**0.92**	0.63	0.67	0.5	0.42	0.56	0.45	0.44	0.7	0.37	0.55
**gCFA**	**0.95**	**0.92**	**1**	**0.98**	0.8	0.68	0.36	0.25	0.44	0.4	0.29	0.73	0.36	0.64
**gEFA**	**0.95**	**0.92**	**0.98**	**1**	0.75	0.65	0.37	0.22	0.54	0.37	0.24	0.74	0.29	0.7
**PMAT24**	0.7	0.63	0.8	0.75	1	0.44	0.14	0.08	0.33	0.28	0.13	0.41	0.18	0.37
**PicVoc**	0.63	0.67	0.68	0.65	0.44	1	0.13	0.15	0.34	0.16	0.15	0.66	0.22	0.29
**CardSort**	0.43	0.5	0.36	0.37	0.14	0.13	1	0.51	0.17	0.18	0.41	0.21	0.12	0.2
**Flanker**	0.35	0.42	0.25	0.22	0.08	0.15	0.51	1	0.11	0.14	0.37	0.15	0.07	0.12
**ListSort**	0.63	0.56	0.44	0.54	0.33	0.34	0.17	0.11	1	0.32	0.16	0.34	0.11	0.24
**PicSeq**	0.52	0.45	0.4	0.37	0.28	0.16	0.18	0.14	0.32	1	0.2	0.17	0.17	0.2
**ProcSpeed**	0.36	0.44	0.29	0.24	0.13	0.15	0.41	0.37	0.16	0.2	1	0.16	0.11	0.14
**ReadVoc**	0.67	0.7	0.73	0.74	0.41	0.66	0.21	0.15	0.34	0.17	0.16	1	0.25	0.35
**WordMem**	0.39	0.37	0.36	0.29	0.18	0.22	0.12	0.07	0.11	0.17	0.11	0.25	1	0.14
**LineOrient**	0.59	0.55	0.64	0.7	0.37	0.29	0.2	0.12	0.24	0.2	0.14	0.35	0.14	1

We found Spearman’s correlations for each variable. Estimates of g are bolded to highlight.

For each of gPCA, gCFA, and gEFA, we investigated pairwise Spearman’s correlations with each of the 91 network reconfiguration variables after regressing out age and gender (Agelink van Rentergem et al., 2020) and FDR corrected across the 91 tests for each estimate of g. We present the results inFigure J.1. In line with Dimension 1 of PLSC, correlations between psychometric g estimates and network reconfiguration metrics ranged from -.14 to .15, indicating a moderate relationship. Additionally, higher transition probability to State 2 and lower transition probability to State 6 were also consistently linked with higher g across psychometric g estimates. However, only gPCA also exhibited positive associations with dwell time and within-state transition probabilities for both States 2 and 3. Apart from minor differences in forward and reverse transitions, similar associations between g and transition distance were also observed across psychometric g estimates. Likewise, consistent associations between g and idiosyncrasy of States 2 through 5 were also identified across psychometric g estimates. This underscores the robustness of our findings, particularly for transition distance and idiosyncrasy.

### Appendix K. Frequency-Based and Distance-Based PLSC

To investigate whether the high correlations observed between traditional frequency-based and novel distance-based metrics mean that PLSC conducted on traditional frequency-based metrics alone would generate the same results, we conducted PLSC on frequency-based metrics and distance-based metrics alone.

For the frequency-based PLSC, we found three significant dimensions (FDR-adjusted*p*< .0001,*p*< .0001,*p = *.0008), but none of the dimensions were fully reproducible. Specifically, none of the SV reproducibility values reached the critical value. Interestingly, the stable loadings for Dimension 1 were ProcSpeed and CardSort, values which were high in Dimension 2 of the original PLSC (Fig. K.1). In fact, there was a positive Spearman’s correlation of .70 between the Dimension 1 cognitive latent variable and the Dimension 2 cognitive latent variable of the original PLSC, and a Spearman’s correlation of .83 between the reconfiguration latent variables of the same dimension. None of the significant dimensions exhibited loadings similar to Dimension 1 of the original PLSC.

For the distance-based PLSC, we found two significant dimensions (FDR-adjusted*p*< .0001), and all of the dimensions were fully reproducible. Interestingly, the loadings resemble Dimension 1 of the original PLSC, though ProcSpeed had a slightly negative loading (Fig. K.1). In fact, there was a Spearman’s correlation of .99 between the Dimension 1 cognitive latent variable and the Dimension 1 cognitive latent variable of the original PLSC, and a Spearman’s correlation of .94 between the reconfiguration latent variables of the same dimension. The second significant dimension also exhibited a stable loading for ProcSpeed, similar to Dimension 2 of the original PLSC, with a Spearman’s correlation of .94 between the Dimension 2 cognitive latent variable and the Dimension 2 cognitive latent variable of the original PLSC, and a Spearman’s correlation of .86 between the reconfiguration latent variables of the same dimensions.

PLSC conducted on distance-based metrics alone produced similar outputs as Dimensions 1 and 2 of the original PLSC, while PLSC conducted on frequency-based metrics alone only produced similar outputs for Dimension 2 of the original PLSC. This suggests that PLSC conducted on frequency-based metrics alone does not generate the same results, in fact, PLSC conducted on distance-based metrics generates much more similar results as the original PLSC. Interestingly, Dimension 1 of the separated PLSCs indicates the distance-based metrics produce the greatest covariance with a latent variable representing g, and frequency-based metrics produce the greatest covariance with a latent variable representing processing speed, seemingly following the observation of a greater number of distance-based loadings in the original Dimension 1 representing the relationship with g and a greater number of frequency-based loadings in the original Dimension 2 representing the relationship with processing speed.
