# Supplementary material for: Higher general intelligence is associated with stable, efficient, and typical dynamic functional brain connectivity patterns
Source: Imaging Neurosci (Camb). 2024 Jul 17;2:imag-2-00234. doi: 10.1162/imag_a_00234 (PMC12272206; doi:10.1162/imag_a_00234)
Supplement: Supplementary Material [file imag_a_00234-supp1.pdf]

Supplemental Material

Supplemental Table 1. Correlations between reconfiguration metrics. Spearman's correlations between each pair of metrics are shown. "Occur" refers to occurrence, "dwell" refers to dwell time, "transnum" refers to transition number, "transpro" refers to transition probability, "transdist" refers to transition distance, and "idio" refers to idiosyncrasy.

Supplemental Table 2. PLSC Diagnostics. Each D refers to a dimension. For the "PLSC - k = 6 Permutation" table, covariance explained is the percentage of total covariance between cognition and network reconfiguration variables explained by the dimension. "SV" is the singular value, "P" is the permutation p-values for the SV, and "Correlation" is the Spearman's correlation between the cognition and network reconfiguration latent variables. Significant ($p < .05$) dimensions are highlighted. For the "PLSC - k = 6 Reproducibility" table, "SV_Z" refers to the Z-value for the SV reproducibility, "LVY_Z" refers to the Z-value for the cognition **Y** latent variable reproducibility, and "LVX_Z" refers to the Z-value for the network reconfiguration **X** latent variable reproducibility. Reproducible ($Z > 1.95$) values are highlighted.

Supplemental Table 3. PLSC relationships between cognition and network reconfiguration metrics. For the "PLSC - k = 6 LVY Loadings" table, "LVY#" refers to the **Y** cognitive test latent variables for each # dimension, and for the "PLSC - k = 6 LVX Loadings" table, "LVX#" refers to the **X** network reconfiguration latent variables for each # dimension. "Load" refers to the loadings, and "boot" refers to the bootstrap ratios. Red highlights stable positive loadings ($|BR| > 2.5$), and blue highlights stable negative loadings. "Occur" refers to occurrence, "dwell" refers to dwell time, "transnum" refers to transition number, "transpro" refers to transition probability, "transdist" refers to transition distance, and "idio" refers to idiosyncrasy.

Supplemental Table 4. Univariate relationships between psychometric g and network reconfiguration metrics. The "gPCA - PC1 Loadings" table contains loadings for each cognitive test for the first principal component (PC1_load) from the PCA, the "gCFA - F1 Loadings table" contains loadings for each cognitive test for the g-factor (F1_load) from the CFA, and the "gEFA - F1 Loadings" table contains loadings for each cognitive test for the g-factor (F1_load) from the CFA. "gPCA - k = 6 Correlations", "gCFA - k = 6 Correlations", and "gEFA - k = 6 Correlations" tables contains the Spearman's correlation (R), *p*-value (P), and FDR-corrected *p*-value (P-Adjusted) with each network reconfiguration variable. Red highlights significant (FDR-corrected $p < .05$) positive values, blue highlights significant negative values. "Occur" refers to occurrence, "dwell" refers to dwell time, "transnum" refers to transition number, "transpro" refers to transition probability, "transdist" refers to transition distance, and "idio" refers to idiosyncrasy.
